# Derivation and Validation of Prediction Models for Prolonged Length of Stay and 30-Day Readmission in Elderly Patients With Type 2 Diabetes Mellitus: A Multicenter Study

**DOI:** 10.1155/jdr/3148242

**Published:** 2025-05-12

**Authors:** Juntao Tan, Yuxin He, Zhengyu Zhang, Jiaxiu Liu, Jinglong Du, Wenlong Zhao, Yanbing Liu

**Affiliations:** ^1^College of Medical Informatics, Chongqing Medical University, Chongqing, China; ^2^Department of Medical Administration, Affiliated Banan Hospital of Chongqing Medical University, Chongqing, China; ^3^Medical Records Department, The First Affiliated Hospital, Zhejiang University School of Medicine, Hangzhou, Zhejiang, China

**Keywords:** 30-day readmission, nomogram, prediction model, prolonged length of stay, Type 2 diabetes mellitus

## Abstract

**Background:** Elderly patients with Type 2 diabetes mellitus (T2DM) often experience prolonged length of stay (LOS) and 30-day readmission. This study was aimed at identifying factors influencing these outcomes and develop predictive models for them.

**Methods:** The least absolute shrinkage and selection operator (LASSO) combined with logistic regression was utilized to construct the prediction models, which were subsequently visualized through nomograms. The performance of these models was comprehensively evaluated in terms of discrimination, calibration, and clinical utility. Specifically, the discrimination capacity was assessed using the area under the receiver operating characteristic curve (AUROC), while calibration was evaluated via calibration curves and the Brier score. Clinical utility was examined through decision curve analysis (DCA) and clinical impact curve (CIC). Additionally, to verify the robustness and generalizability of the developed prediction models, subgroup analyses were conducted across various strata of the study population.

**Results:**A total of 24 variables for 8800 patients were included for predicting prolonged LOS, and 38 variables were used for 30-day readmission prediction. In the training set, 28.42% of patients had prolonged LOS and 13.68% were readmitted within 30 days. The prolonged LOS model had an AUROC of 0.720 (95% CI: 0.703–0.737), while the 30-day readmission model achieved 0.766 (95% CI: 0.745–0.787). The Brier scores were 0.174 (95% CI: 0.168–0.180) and 0.102 (95% CI: 0.096–0.108), respectively. Both models showed good clinical utility in DCA and CIC analyses. Subgroup validation across different age groups showed consistent performance, with all AUROCs above 0.60. Albumin was identified as the most significant predictor in both models.

**Conclusion:** The predictive models developed in this study demonstrated robust performance in forecasting common outcomes in elderly patients with T2DM. Moreover, albumin level was strongly associated with both prolonged LOS and 30-day readmission, making it a key factor in patient management.

## 1. Introduction

The global prevalence of diabetes has risen significantly in recent years, resulting in increased healthcare costs and placing a substantial burden on healthcare systems worldwide [1, 2]. According to the International Diabetes Federation (IDF), approximately 10.5% of the global population aged 20–79 (536.6 million people) had diabetes in 2021, and this number is expected to rise to 12.2% (783.2 million people) by 2045 [3]. In China, the prevalence of Type 2 diabetes mellitus (T2DM) increased from 10.9% in 2012 to 12.4% in 2018 [4]. The economic impact of diabetes is equally concerning, with global healthcare expenditures projected to reach $1054 billion by 2045 [3]. In 2014, China recorded the highest direct medical costs for diabetes globally, totaling $170 billion [5]. By 2020, these costs had risen to $190.2 billion, with per capita economic burdens reaching $231. If current trends continue, these figures are expected to increase to $337.8 billion and $414, respectively, by 2030, with annual growth rates of 5.98% and 6.02% [6].

Prolonged length of stay (LOS) and 30-day readmission are critical indicators of patient health outcomes and healthcare efficiency [7, 8]. Prolonged LOS is associated with adverse health effects, including depression [9] and an increased risk of hospital-acquired infections [10–12], often leading to a cycle of delayed discharges [13]. Similarly, 30-day readmission rates are widely used as a measure of hospital performance and quality of care, reflecting potential gaps in patient management [14, 15]. Research shows that diabetes patients have significantly higher 30-day readmission rates compared to the general hospitalized population [16, 17]. Both prolonged LOS and 30-day readmission contribute to rising healthcare costs, particularly among elderly patients. These issues highlight the growing economic and clinical burden of T2DM in elderly populations, necessitating urgent attention and intervention.

Despite the significance of these outcomes, there is limited research focusing on prolonged LOS and 30-day readmission among elderly T2DM patients. This study is aimed at addressing this gap by identifying risk factors associated with prolonged LOS and 30-day readmissions in this population. Additionally, we aim to develop predictive models to facilitate early identification of high-risk patients, enabling the implementation of targeted interventions to improve outcomes and reduce healthcare costs.

## 2. Methods

### 2.1. Data Source and Study Participants

This study was approved by the ethics committee of the Chongqing Medical University (Approval No. 2024097), the ethics committee of the Affiliated Banan Hospital of Chongqing Medical University (Approval No. BNLLKY2023037), and the ethics committee of the First Affiliated Hospital of Zhejiang University School of Medicine (Approval No. IIT20230312B-R1). Due to the retrospective nature of the study, informed consent was waived, and the research was conducted in accordance with national regulations and institutional guidelines.

**Figure 1:**
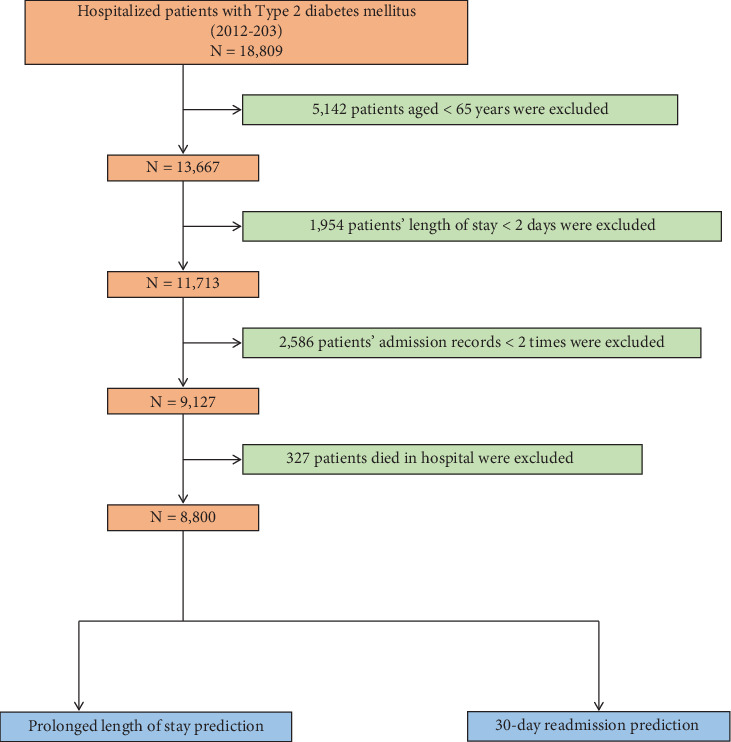
Patient flow diagram.

To ensure scientific rigor, internal consistency, clinical relevance, and data quality, we established the following inclusion and exclusion criteria. Inclusion criteria were (1) hospitalization(s) for T2DM and (2) data collected between 2012 and 2023. Exclusion criteria were (1) age < 65 years (*n* = 5142), (2) LOS < 2 days (*n* = 1954), (3) fewer than two admission records (*n* = 2586), and (4) in-hospital death (*n* = 327). After applying these criteria, 8800 patients were included in the study. The patient selection process is summarized in Figure 1.

Data were obtained from six tertiary hospitals affiliated with the Medical Data Science Academy of Chongqing Medical University, covering the period from January 2011 to December 2022. Patients from these hospitals were randomly divided into a training set (*n* = 4729) and an internal validation set (*n* = 2027) in a 7:3 ratio. Additionally, patients from the Affiliated Banan Hospital of Chongqing Medical University (January 2018–December 2023, *n* = 1244) and the First Affiliated Hospital of Zhejiang University School of Medicine (January 2020–December 2022, *n* = 800) were used as External Validation Set I and External Validation Set II, respectively.

### 2.2. Predictors and Outcomes

Clinical data were extracted from the electronic medical record (EMR) system within 48 h of admission: (1) basic information: age, sex, insurance type, past surgical history (PSH), smoking history, drinking history, age-adjusted Charlson comorbidity index (ACCI) score, and LOS; (2) diagnosis: hypertension, coronary heart disease (CHD), cerebral infarction (CI), and hyperlipidemia; (3) laboratory measurements: aspartate aminotransferase (AST), alanine aminotransferase (ALT), triglycerides (TGs), creatinine (CREA), uric acid (UA), low-density lipoprotein cholesterol (LDL-C), high-density lipoprotein cholesterol (HDL-C), albumin (ALB), estimated glomerular filtration rate (eGFR), neutrophil-to-lymphocyte ratio (NLR), platelet-to-lymphocyte ratio (PLR), lymphocyte-to-monocyte ratio (LMR), and neutrophil percentage-to-albumin ratio (NPAR); (4) vital signs: systolic blood pressure (SBP) and diastolic blood pressure (DBP); (5) medication administration: nutritional support drug use, antihypertensive drug use, antidiabetic drug use, statin drug use, antiplatelet and anticoagulant drug use, Chinese patent medicine use, analgesic drug use, antiosteoporotic drug use, antibiotic drug use, hemostatic agent drug use, and psychotropic drug use.

This study focused on two main outcomes: prolonged LOS and 30-day readmission. Prolonged LOS was defined as a binary variable, with the cutoff set at the 75th percentile of the LOS distribution among participants, which was 14 days [18–20]. Patients with a LOS of 14 days or more were coded as 1, while those with less than 14 days were coded as 0. A 30-day readmission was defined as any readmission occurring within 30 days of discharge, regardless of the reason. The time to readmission was calculated by subtracting the LOS of the initial admission from the interval between the two admissions. Only the first readmission and its corresponding initial admission were included in the final analysis.

### 2.3. Definition

The type of insurance selected by individuals reflects the economic burden on them and their families. In this study, insurance types were categorized as urban employee medical insurance (UEMI), urban resident medical insurance (URMI), full self-payment, and other insurance. The ACCI score was used to quantify comorbidities based on the number and severity of illnesses, serving as a prognostic evaluation tool [21]. The eGFR was calculated using the Modification of Diet in Renal Disease (MDRD) study equations [22], in line with current recommendations. Novel markers such as the NLR, PLR, LMR, and NPAR were used to assess systemic inflammation and have been linked to adverse outcomes [23–25].

### 2.4. Model Development

Variables identified as statistically significant in univariate logistic regression analysis were included in the least absolute shrinkage and selection operator (LASSO) regression analysis, followed by a multivariate logistic regression model. A nomogram was then constructed using the “rms” package in R. Each regression coefficient from the multivariate logistic regression model was proportionally converted into a score ranging from 0 to 100, with the highest absolute *β* coefficient assigned a score of 100. The total score was calculated by summing the scores of each variable, and this total score was then converted into a predicted probability.

### 2.5. Model Performance

To avoid overfitting and determine the optimal number of variables for the model, we iteratively identified *K* variables associated with prolonged LOS and 30-day readmission. We plotted the mean area under the receiver operating characteristic curve (AUROC) for different values of *K* to ensure a robust model. For model evaluation, we calculated the AUROC for the training set, internal validation set, and external validation sets. We also assessed sensitivity, specificity, positive predictive value (PPV), negative predictive value (NPV), and Youden's index. Calibration plots and Brier scores were used to illustrate the relationship between predicted risk values and actual observed outcomes. Decision curve analysis (DCA) and clinical impact curve (CIC) were performed to evaluate the clinical effectiveness of the model and quantify the net benefit to patients. Subgroup analysis was conducted by categorizing patients into three age groups: under 75 years, 75–84 years, and over 84 years, to assess the model's performance within each subgroup.

### 2.6. Statistical Analyses

We followed the transparent reporting of a multivariable prediction model for individual prognosis or diagnosis (TRIPOD) guidelines for reporting (Table S1) [26]. Missing data were handled using multivariate multiple imputation with chained equations to maximize statistical power and minimize bias. Discrete variables were expressed as rates and percentages, with the chi-square test used for group comparisons. Quantitative variables, which did not follow a normal distribution, were represented by the median and interquartile range [*M*(*Q*25 − *Q*75)], and the Mann–Whitney *U* test was used for group comparisons. All statistical analyses were two-sided, with statistical significance set at *p* < 0.05. Analyses were performed using SPSS Version 22.0 and R (Version 4.3.2, Vienna, Austria).

## 3. Results

### 3.1. Patient Characteristics

A total of 8800 patients were included in the study, divided into four groups: 4729 in the training set, 2027 in the internal validation set, 1244 in External Validation Set I, and 800 in External Validation Set II. These groups were selected for predicting prolonged LOS and 30-day readmission after excluding individuals who did not meet the inclusion criteria. In the training and internal validation sets, 1902 patients (28.15%) experienced prolonged LOS, and 923 patients (13.66%) were readmitted within 30 days of discharge. Detailed patient characteristics, stratified by outcomes, are presented in Tables 1 and 2. The Mann–Whitney *U* test showed no significant differences in missing variables between the training and internal validation sets before and after multiple imputations (Table S2).

Patients with prolonged LOS were more likely to be male, have a history of smoking and drinking, and be diagnosed with hypertension, CHD, and CI. They also exhibited higher levels of AST, ALT, NLR, PLR, NPAR, and CREA. Conversely, they had lower levels of TGs, LMR, UA, LDL-C, HDL-C, ALB, and GFR. Patients readmitted within 30 days were younger, more likely to be male, had a longer LOS, and were more likely to have UEMI. They also had a higher prevalence of smoking, drinking, and higher ACCI scores. These patients were more frequently diagnosed with CHD, CI, and hyperlipidemia and were more likely to use nutritional support drugs, antidiabetic drugs, statins, antiplatelet and anticoagulant drugs, Chinese patent medicines, analgesics, antibiotics, hemostatic agents, and psychotropic drugs. They exhibited higher levels of NLR, PLR, NPAR, and TGs but lower SBP, TGs, LMR, LDL-C, HDL-C, and ALB.

### 3.2. Selection of Predictors for Prolonged LOS and 30-Day Readmission

In the univariate analysis, several factors were significantly associated with prolonged LOS, including sex, smoking history, drinking history, hypertension, CHD, CI, PSH, AST, ALT, TGs, NLR, LMR, PLR, NPAR, CREA, UA, LDL-C, HDL-C, ALB, and GFR (*p* < 0.05). Similarly, factors significantly associated with 30-day readmission included age, sex, LOS, smoking history, drinking history, insurance type, ACCI score, CHD, CI, hyperlipidemia, and the use of various medications (*p* < 0.05) (Tables 3 and 4).

**Figure 2:**
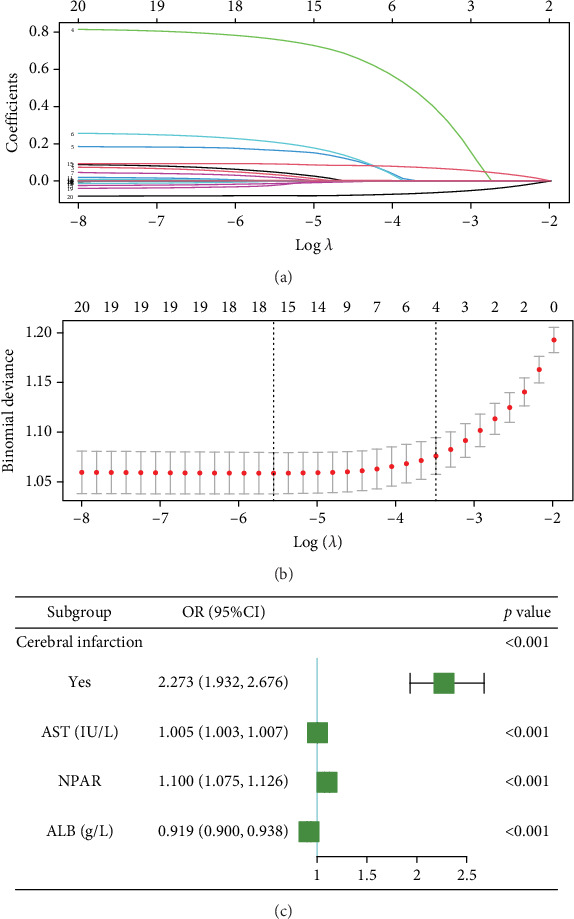
The screening results of predictors for prolonged LOS. (a) LASSO coefficient profiles (*y*-axis) of features. The upper *x*-axis is the average number of predictors and the lower *x*-axis is the log(*λ*). (b) Tenfold cross-validation for tuning parameter selection in the LASSO model. The dotted vertical lines were drawn at optimal values by using the minimum criteria and within one standard error range of the minimum criteria. In the LASSO model, we initially conducted 10-fold cross-validation of LASSO to select candidate variables (“glmnet” package). (c) Forest plot showing the results of multivariable analysis for prolonged LOS and 30-day readmission.

**Figure 3:**
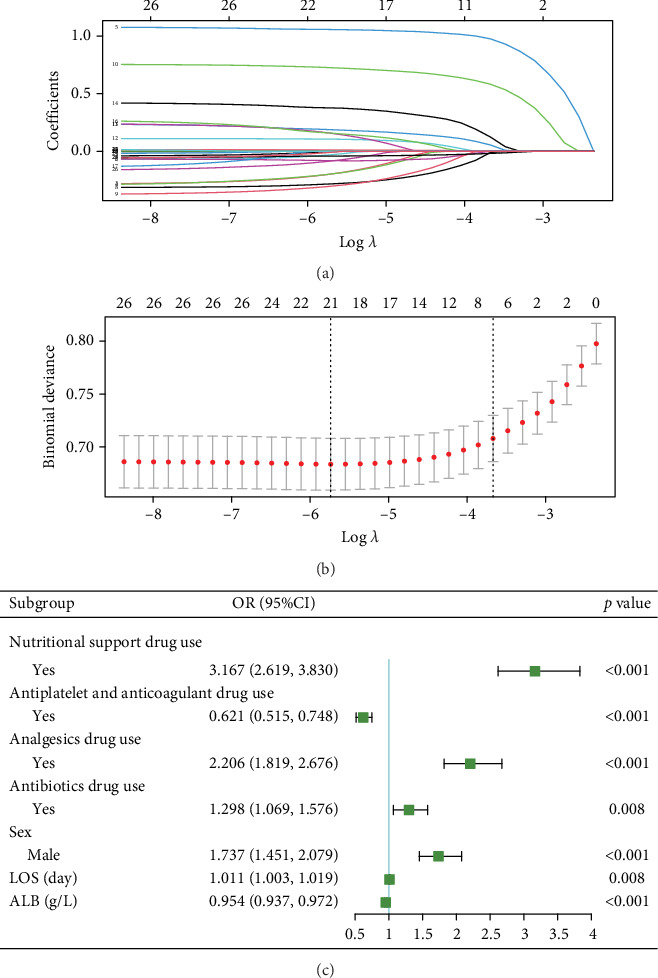
The screening results of predictors for 30-day readmission. (a) LASSO coefficient profiles (*y*-axis) of features. The upper *x*-axis is the average number of predictors and the lower *x*-axis is the log(*λ*). (b) Tenfold cross-validation for tuning parameter selection in the LASSO model. The dotted vertical lines were drawn at optimal values by using the minimum criteria and within one standard error range of the minimum criteria. In the LASSO model, we initially conducted 10-fold cross-validation of LASSO to select candidate variables (“glmnet” package). (c) Forest plot showing the results of multivariable analysis for prolonged LOS and 30-day readmission.

For prolonged LOS, 20 variables were entered into the LASSO regression analysis, and four were identified as significant predictors: CI, AST, NPAR, and ALB (Figure 2a,b). The final multivariate logistic regression model confirmed that CI (OR: 2.273, 95% CI: 1.932–2.676, *p* < 0.001), AST (OR: 1.005, 95% CI: 1.003–1.007, *p* < 0.001), NPAR (OR: 1.100, 95% CI: 1.075–1.126, *p* < 0.001), and ALB (OR: 0.919, 95% CI: 0.900–0.938, *p* < 0.001) were independent predictors of prolonged LOS (Figure 2c). For 30-day readmission, seven predictors were identified: sex, LOS, nutritional support drug use, antiplatelet and anticoagulant drug use, analgesic drug use, antibiotic drug use, and ALB (Figure 3a,b). The multivariate logistic regression model showed that sex (OR: 1.737, 95% CI: 1.451–2.079, *p* < 0.001), LOS (OR: 1.011, 95% CI: 1.003–1.019, *p* < 0.001), nutritional support drug use (OR: 3.167, 95% CI: 2.619–3.830, *p* < 0.001), antiplatelet and anticoagulant drug use (OR: 0.621, 95% CI: 0.515–0.748, *p* < 0.001), analgesic drug use (OR: 2.201, 95% CI: 1.819–2.676, *p* < 0.001), antibiotic drug use (OR: 1.298, 95% CI: 1.069–1.576, *p* = 0.008), and ALB (OR: 0.954, 95% CI: 0.937–0.972, *p* < 0.001) were independent predictors of 30-day readmission (Figure 3c).

**Figure 4:**
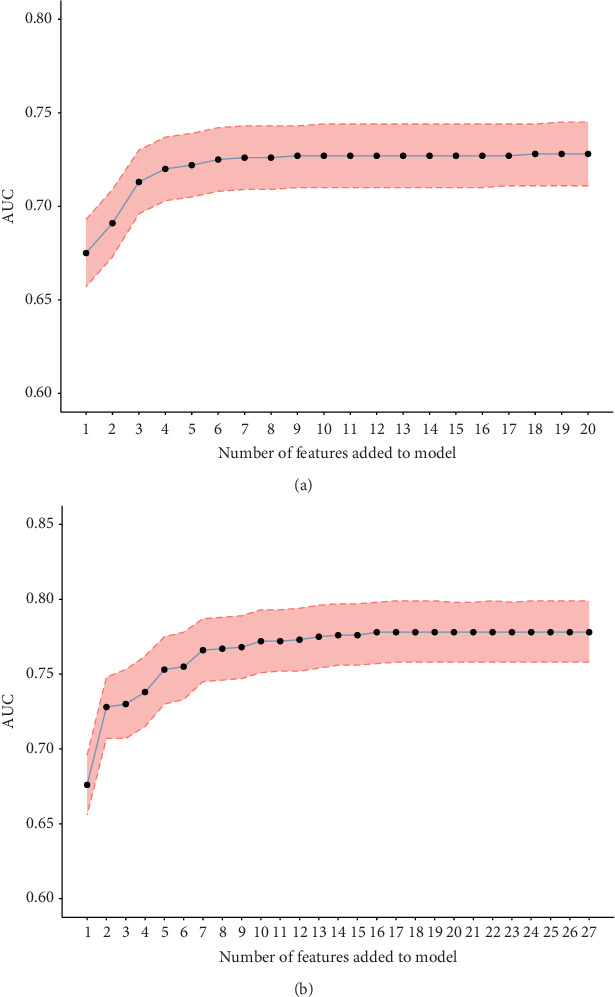
Identification of the optimal numbers of variables for prediction of prolonged LOS and 30-day readmission. (a) Prolonged LOS; (b) 30-day readmission.

To validate the performance of the LASSO-logistic regression, we evaluated subsets of variables associated with prolonged LOS and 30-day readmission using the top *K* features. The addition of more variables beyond four for prolonged LOS and seven for 30-day readmission did not significantly improve the AUROC (Figure 4). This suggests that including additional variables, even if closely related to the outcomes, may not enhance the predictive performance of the models (Table S3).

### 3.3. Development and Validation of the Prolonged LOS Model

**Figure 5:**
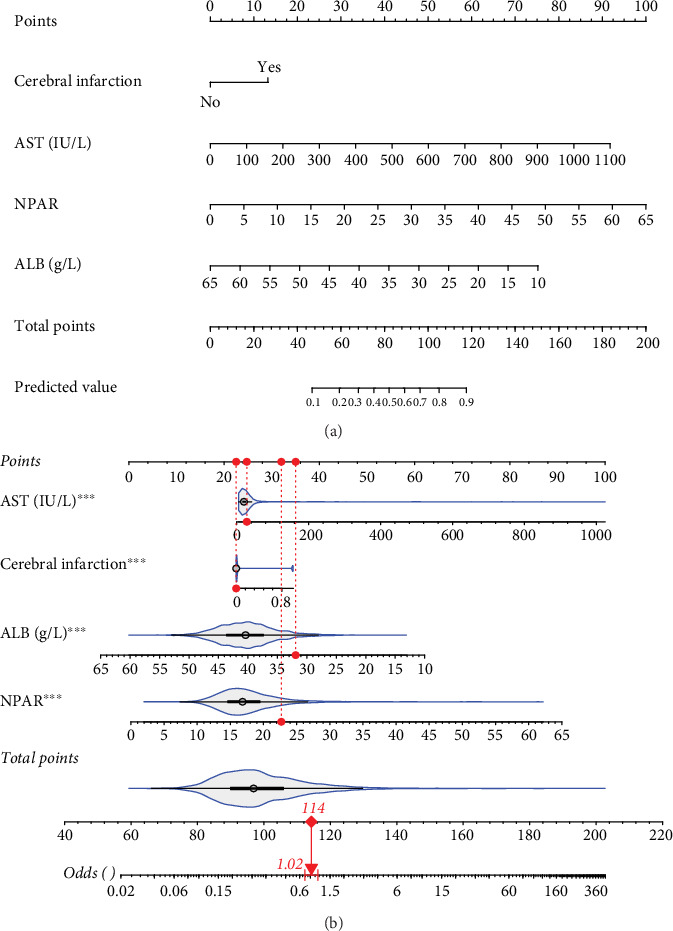
Nomogram for the prolonged LOS model. (a) Nomogram to estimate the risk of prolonged LOS in elderly patients with T2DM. (b) One patient from our study is shown as an example (presented in red).

A static nomogram was developed to predict prolonged LOS based on a four-predictor model (Figure 5a).  Figure 5b demonstrates how the nomogram can be used to estimate the probability of prolonged LOS for a patient in the training set. The total score, derived from the predictors, provides the predicted probability. Additionally, a dynamic nomogram was created and made available online, allowing clinicians to calculate the probability of prolonged LOS in elderly patients with T2DM (https://cqmuhyx.shinyapps.io/prolonged_los/).

**Figure 6:**
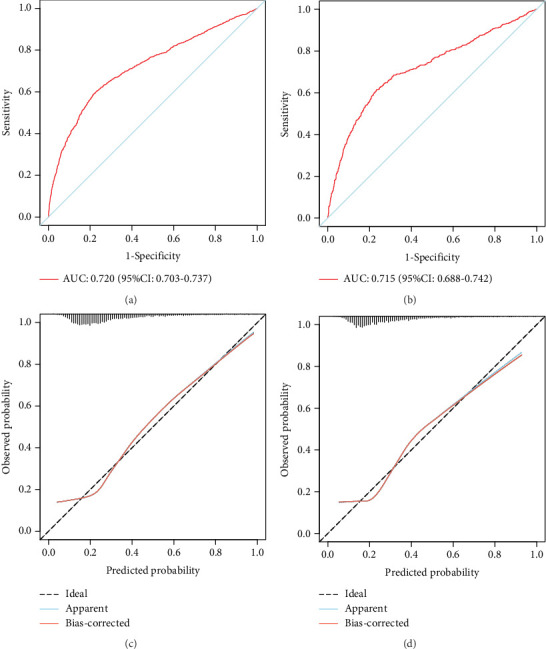
Comprehensive evaluation of the prolonged LOS model. (a, b) The ROC curves of the model in training and internal validation sets. (c, d) The calibration curves of the model in training and internal validation sets. The diagonal dotted line indicates the best prediction by an ideal model. The apparent line represents the uncorrected performance of the nomogram, and the red line shows the bias-corrected performance.

The model showed strong predictive performance, with AUROC values of 0.720 (95% CI: 0.703–0.737) in the training set, 0.715 (95% CI: 0.688–0.742) in the internal validation set, 0.736 (95% CI: 0.696–0.777) in External Validation Set I, and 0.703 (95% CI: 0.645–0.761) in External Validation Set II (Figure 6a,b; Figure S1A, B). The optimal decision probability cutoff was 0.311. Calibration curves indicated strong agreement between predicted and actual probabilities in the training set (Brier score = 0.174, 95% CI: 0.168–0.180), internal validation set (*Brier* *score* = 0.172, 95% CI: 0.163–0.182), External Validation Set I (Brier score = 0.123, 95% CI: 0.110–0.136), and External Validation Set II (Brier score = 0.106, 95% CI: 0.090–0.121) (Figure 6c,d; Figure S1C, D). Overall, the nomogram demonstrated excellent discrimination and calibration. Detailed performance metrics for all datasets are provided in Table 5.

DCA showed favorable clinical utility. Specifically, when the threshold probability exceeded 12%, using the model to predict prolonged LOS and guide interventions provided greater net benefit than treating all or no patients (Figure S2A). The CIC further confirmed the model's superior net benefit within the threshold probability range (Figure S2C). DCAs and CICs for the internal and external validation sets are shown in Figure S2B, S1E, F and Figure S2D, S1G, H, respectively.

### 3.4. Development and Validation of the 30-Day Readmission Model

**Figure 7:**
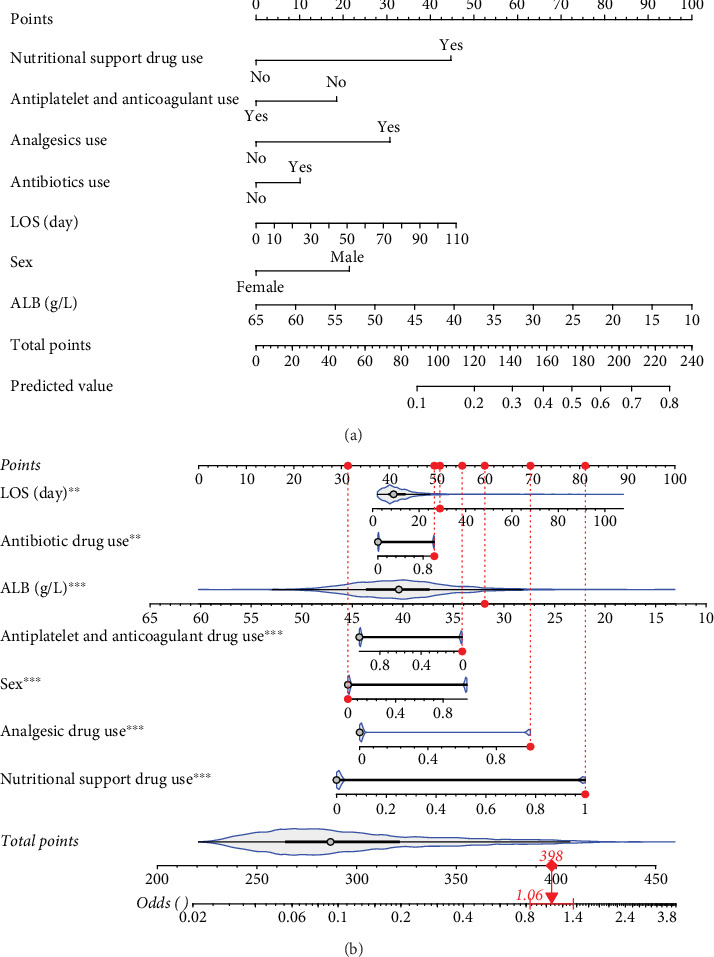
Nomogram for the 30-day readmission model. (a) Nomogram to estimate the risk of prolonged LOS in elderly patients with T2DM. (b) One patient from our study is shown as an example (presented in red).

A nomogram was developed to predict 30-day readmission in elderly patients with T2DM (Figure 7a).  Figure 7b illustrates its application for a patient in the training set. A user-friendly web interface (https://cqmuhyx.shinyapps.io/readmission/) was also created to help clinicians calculate the 30-day readmission probability.

**Figure 8:**
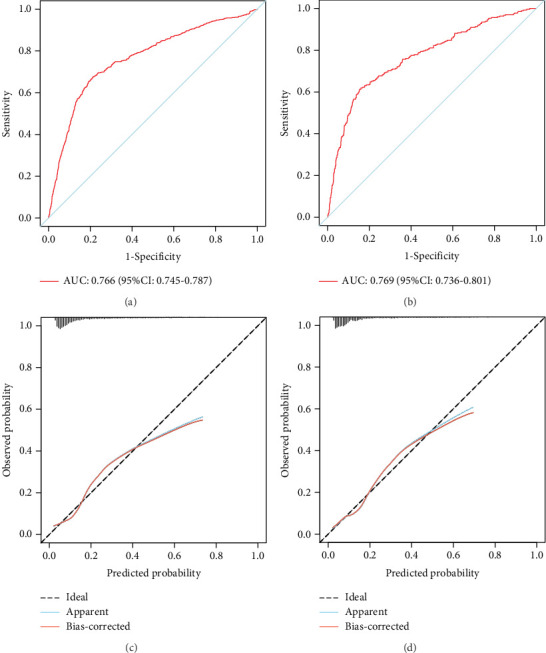
Comprehensive evaluation of the 30-day readmission model. (a, b) The ROC curves of the model in training and internal validation sets. (c, d) The calibration curves of the model in training and internal validation sets. The diagonal dotted line indicates the best prediction by an ideal model. The apparent line represents the uncorrected performance of the nomogram, and the red line shows the bias-corrected performance.

The model demonstrated strong predictive performance, with AUROC values of 0.766 (95% CI: 0.745–0.787) in the training set, 0.769 (95% CI: 0.736–0.801) in the internal validation set, 0.633 (95% CI: 0.594–0.673) in External Validation Set I, and 0.671 (95% CI: 0.613–0.729) in External Validation Set II (Figures 3a,b and 8a,b). The optimal decision probability cutoff was 0.148. Calibration curves for the training set (Brier score = 0.102, 95% CI: 0.096–0.108), internal validation set (Brier score = 0.100, 95% CI: 0.090–0.101), External Validation Set I (Brier score = 0.156, 95% CI: 0.143–0.169), and External Validation Set II (Brier score = 0.093, 95% CI: 0.078–0.109) confirmed the nomogram's accuracy (Figure 8c,d; Figure S3C, D). Detailed performance metrics are provided in Table 6.

DCA (Figure S4A) and CIC (Figure S4C) confirmed the clinical utility of the nomogram. The DCA showed that the nomogram provided greater net benefit when the high-risk threshold probability exceeded 10%. The CIC, applied to a sample size of 1000, demonstrated that the predicted number of 30-day readmissions closely matched the actual number when the threshold probability was above 0.40. DCAs and CICs for the internal and external validation sets are shown in Figure S4B, S3E, F and Figure S4D, S3E, F, respectively.

### 3.5. Subgroup Analysis by Age

The prolonged LOS and 30-day readmission prediction models demonstrated good discrimination across all age subgroups, with AUROCs exceeding 0.60 in all groups (Table 7). Sensitivity, specificity, NPV, PPV, and Brier scores for each age subgroup are provided in Tables S4–S8. Figures S5–S12 show the ROC curves, calibration curves, decision curves, and CICs for all age subgroups. These results confirm the robustness of the models across different age groups.

## 4. Discussion

Using a comprehensive multicenter database, we developed and validated two predictive models to identify prolonged LOS and 30-day readmission in elderly patients with T2DM. The large sample size and diverse hospital network ensure the statistical robustness and broad clinical applicability of our models.

This study focused on predicting outcomes in elderly T2DM patients without distinguishing other comorbidities. The observed prolonged LOS rate of 28.15% and 30-day readmission rate of 13.66% are consistent with previous studies [13, 27]. To enhance clinical utility, we included only commonly used and easily accessible variables collected within 48 h from EMRs. This resulted in fewer variables compared to similar studies, such as Collins et al.'s study [28] with nearly 200 variables and Sherman et al.'s study [29] with 165 variables. In contrast, our study incorporated only 38 clinically relevant and easily obtainable variables. Despite this, the models demonstrated good discriminative ability, with AUROC values of 0.720 (95% CI: 0.703–0.737) for prolonged LOS and 0.766 (95% CI: 0.745–0.787) for 30-day readmission.

Our findings identified CI (OR: 2.273, 95% CI: 1.932–2.676, *p* < 0.001) as an independent risk factor for prolonged LOS in elderly T2DM patients. Epidemiological studies have shown that T2DM is associated with a two- to fivefold increased risk of ischemic cerebrovascular disease, particularly CI [30–32]. A longitudinal study also linked T2DM to a higher risk of CI, which contributes to prolonged LOS [33]. NPAR has emerged as a novel inflammatory biomarker and has been investigated as an independent prognostic marker in various clinical settings, including N-methyl-D-aspartate receptor encephalitis [34], malignant tumors [35], and other diseases [36]. Our study further supports the prognostic value of NPAR, demonstrating that it is a significant risk factor for prolonged LOS in elderly patients with T2DM (OR: 1.100, 95% CI: 1.075–1.126, *p* < 0.001). NPAR is simple, cost-effective, and easily accessible, making it a valuable tool for clinicians, especially when neutrophil percentage and ALB levels are within normal ranges. AST (OR: 1.005, 95% CI: 1.003–1.007, *p* < 0.001) was also identified as a risk factor, as elevated AST levels often indicate cellular damage due to reduced blood supply or oxygenation [37].

For 30-day readmission, the use of nutritional support drugs, analgesics, and antibiotics was significantly associated with higher readmission rates, reflecting overall disease burden. A local study found that the number of medications was a predictive factor for readmission [38], and medication nonadherence due to side effects may further explain this association [39]. Interestingly, antiplatelet and anticoagulant drug use was negatively correlated with 30-day readmission (OR: 0.621, 95% CI: 0.515–0.748, *p* < 0.001), likely because these drugs help prevent cardiovascular events [40], a common cause of readmission in T2DM patients [41].

Consistent with previous studies by Collins et al. [28] and Regassa and Tola [42], we found that male sex was a significant risk factor for 30-day readmission, with males having 1.74 times higher odds (95% CI: 1.45–2.08) compared to females. Additionally, longer LOS was associated with a higher risk of 30-day readmission (OR: 1.011, 95% CI: 1.003–1.019, *p* < 0.001). LOS reflects disease severity, and prolonged hospitalization increases the risk of hospital-acquired infections, further elevating readmission risk [27, 43].

Interestingly, ALB was the only factor significantly associated with both prolonged LOS (OR: 0.919, 95% CI: 0.900–0.938, *p* < 0.001) and 30-day readmission (OR: 0.954, 95% CI: 0.937–0.972, *p* < 0.001). Low ALB levels can result from decreased synthesis, increased catabolism, heightened vascular permeability, or renal loss. Malnutrition and inflammation are the primary drivers of hypoalbuminemia [44–46]. ALB plays a multifaceted role in health and disease, serving as a marker of nutritional status and a regulator of inflammation, oxidative stress, and endothelial function. Low ALB levels often indicate systemic inflammation and chronic disease severity, which are linked to prolonged hospitalization and higher readmission rates [47–49]. For instance, hypoalbuminemia is associated with impaired immune response, delayed wound healing, and increased infection risk, all of which can extend hospital stays and raise readmission likelihood [50, 51].

The normal reference range for ALB in adults is typically 3.5–5.0 g/dL. A cohort study found that patients with higher ALB levels had a lower risk of death and readmission (HR: 0.78, *p* < 0.001) [52]. Another study showed that higher ALB levels were associated with a reduced risk of microvascular complications in diabetes patients [53]. Specifically, for every 10 g/L increase in ALB, the HRs for diabetic nephropathy, ophthalmopathy, and neuropathy were 0.42 (95% CI: 0.30–0.58), 0.61 (95% CI: 0.52–0.72), and 0.67 (95% CI: 0.51–0.88), respectively. These findings highlight ALB's importance as a biomarker for both short- and long-term outcomes in diabetes. This partly explains ALB's influence on prolonged LOS and 30-day readmission. Therefore, ALB should be included in routine admission tests for elderly T2DM patients. If ALB levels are below normal, appropriate interventions, such as nutritional support or anti-inflammatory therapies, should be considered. Future studies should investigate whether improving ALB levels can reduce prolonged hospitalization and readmission risks in this population.

In addition to the findings of our study, the potential role of SGLT2 inhibitors in preventing adverse events, such as frailty, in older adults with diabetes warrants further discussion. Recent studies have demonstrated that SGLT2 inhibitors, such as empagliflozin, not only improve glycemic control but also offer significant benefits in reducing the risk of cardiovascular events, slowing the progression of chronic kidney disease, and potentially mitigating cognitive and physical decline in frail older adults [54–56]. These findings highlight the functional and clinical importance of SGLT2 inhibitors in this vulnerable population, particularly in the context of multimorbidity and polypharmacy [57–60]. Future research should explore the optimal use of SGLT2 inhibitors in older adults with diabetes, taking into account their level of frailty and comorbidities.

This study has two main strengths: first, the use of a large, multicenter dataset to develop the predictive models; second, the inclusion of simple, easily obtainable variables, which enhances the models' generalizability and clinical applicability. However, limitations must be acknowledged. First, the retrospective design provides weaker evidence compared to prospective studies, so findings should be interpreted cautiously. Second, some potential predictors were excluded due to high rates of missing data, which may have affected the models' predictive performance. Future prospective studies with more comprehensive data and larger sample sizes are needed to validate or refine these findings.

## 5. Conclusion

Two predictive models were developed to assess prolonged LOS and 30-day readmission in elderly T2DM patients. Both models demonstrated strong performance, with AUROC values of 0.720 for prolonged LOS and 0.766 for 30-day readmission. The inclusion of inexpensive and readily available variables, such as demographic characteristics, comorbidities, and routine laboratory results, enhances the models' feasibility for clinical use. These models offer valuable tools for identifying high-risk patients and guiding targeted interventions to improve outcomes.

## Figures and Tables

**Table 1:** Baseline characteristics for prolonged LOS in the training and internal validation sets.

**Variables**	**Total (** **N** = 6756**)**	**Prolonged LOS (** **N** = 1902**)**	**Normal LOS (** **N** = 4854**)**	**p** ** value**
*Basic information*				
Age (year)	74.00 (69.00, 79.00)	74.00 (69.00, 80.00)	73.00 (69.00, 79.00)	0.069
Sex (*n*, %)				< 0.001
Female	3744 (55.42)	960 (50.47)	2784 (57.35)	
Male	3012 (44.58)	942 (49.53)	2070 (42.65)	
PSH (*n*, %)				0.051
Yes	4488 (66.43)	1298 (68.24)	3190 (65.72)	
No	2268 (33.57)	604 (31.76)	1664 (34.28)	
Smoking history (*n*, %)				< 0.001
Yes	1960 (29.01)	614 (32.28)	1346 (27.73)	
No	4796 (70.99)	1288 (67.72)	3508 (72.27)	
Drinking history (*n*, %)				0.029
Yes	1585 (23.46)	481 (25.29)	1104 (22.74)	
No	5171 (76.54)	1421 (74.71)	3750 (77.26)	
*Diagnosis*				
Hypertension (*n*, %)				< 0.001
Yes	4350 (64.39)	1287 (67.67)	3063 (63.10)	
No	2406 (35.61)	615 (32.33)	1791 (36.90)	
CHD (*n*, %)				0.010
Yes	2223 (32.90)	671 (35.28)	1552 (31.97)	
No	4533 (67.10)	1231 (64.72)	3302 (68.03)	
CI (*n*, %)				< 0.001
Yes	1221 (18.07)	514 (27.02)	707 (14.57)	
No	5535 (81.93)	1388 (72.98)	4147 (85.43)	
Hyperlipidemia (*n*, %)				0.247
Yes	1026 (15.19)	273 (14.35)	753 (15.51)	
No	5730 (84.81)	1629 (85.65)	4101 (84.49)	
*Laboratory measurements*				
AST (IU/L)	20.00 (16.00, 26.00)	20.35 (16.00, 28.50)	19.95 (16.00, 25.00)	< 0.001
ALT (IU/L)	18.00 (13.00, 26.00)	18.00 (13.00, 29.00)	17.60 (13.00, 25.00)	< 0.001
TGs (mmol/L)	1.42 (1.04, 2.04)	1.39 (1.00, 1.96)	1.44 (1.06, 2.08)	< 0.001
NLR	2.98 (2.08, 4.74)	3.56 (2.33, 6.11)	2.81 (2.01, 4.26)	< 0.001
LMR	3.88 (2.59, 5.48)	3.33 (2.10, 4.87)	4.11 (2.82, 5.68)	< 0.001
PLR	124.38 (91.01, 172.37)	132.21 (95.49, 191.60)	122.00 (90.00, 164.85)	< 0.001
NPAR	16.79 (14.63, 19.44)	18.83 (15.63, 22.40)	16.31 (14.35, 18.45)	< 0.001
CREA (*μ*mol/L)	72.00 (57.90, 92.10)	75.40 (59.10, 99.50)	71.10 (57.50, 89.40)	< 0.001
UA (*μ*mol/L)	326.25 (260.58, 399.00)	316.10 (254.05, 397.58)	328.60 (262.89, 399.25)	0.011
LDL-C (mmol/L)	2.49 (1.90, 3.14)	2.35 (1.78, 3.00)	2.53 (1.95, 3.20)	< 0.001
HDL-C (mmol/L)	1.12 (0.93, 1.35)	1.08 (0.88, 1.32)	1.14 (0.95, 1.36)	< 0.001
ALB (g/L)	40.60 (37.50, 43.60)	38.01 (34.30, 41.74)	41.24 (38.60, 44.10)	< 0.001
GFR (mL/min)	83.55 (63.03, 101.95)	81.06 (57.68, 100.47)	84.33 (64.94, 102.65)	< 0.001
*Vital signs*				
SBP (mmHg)	139.00 (126.00, 154.00)	139.00 (125.00, 154.00)	140.00 (126.00, 153.00)	0.605
DBP (mmHg)	78.00 (70.00, 85.00)	78.00 (70.00, 85.00)	78.00 (70.00, 85.00)	0.274

Abbreviations: ALB, albumin; ALT, alanine aminotransferase; AST, aspartate aminotransferase; CHD, coronary heart disease; CI, cerebral infarction; CREA, creatinine; DBP, diastolic blood pressure; eGFR, estimated glomerular filtration rate; HDL-C, high-density lipoprotein cholesterol; LDL-C, low-density lipoprotein cholesterol; LMR, lymphocyte-to-monocyte ratio; NLR, neutrophil-to-lymphocyte ratio; NPAR, neutrophil percentage-to-albumin ratio; PLR, platelet-to-lymphocyte ratio; PSH, past surgical history; SBP, systolic blood pressure; TGs, triglycerides; UA, uric acid.

**Table 2:** Baseline characteristics for 30-day readmission in the training and internal validation sets.

**Variables**	**Total (** **N** = 6756**)**	**30-day readmission (** **N** = 923**)**	**Non-30-day readmission (** **N** = 5833**)**	**p** ** value**
*Basic information*				
Age (year)	74.00 (69.00, 79.00)	73.00 (68.00, 78.00)	74.00 (69.00, 79.00)	0.003
Sex (*n*, %)				< 0.001
Female	3744 (55.42)	389 (42.15)	3355 (57.52)	
Male	3012 (44.58)	534 (57.85)	2478 (42.48)	
Insurance type (*n*, %)				0.005
Full self-pay	590 (8.73)	66 (7.15)	524 (8.98)	
UEMI	4599 (68.07)	649 (70.31)	3950 (67.72)	
URMI	1250 (18.51)	182 (19.72)	1068 (18.31)	
Other insurance	317 (4.69)	26 (2.82)	291 (4.99)	
ACCI score (*n*, %)				0.003
< 8	1792 (26.52)	282 (30.55)	1510 (25.89)	
≥ 8	4964 (73.48)	641 (69.45)	4323 (74.11)	
PSH (*n*, %)				0.240
Yes	4488 (66.43)	597 (64.68)	3891 (66.71)	
No	2268 (33.57)	326 (35.32)	1942 (33.29)	
Smoking history (*n*, %)				< 0.001
Yes	1960 (29.01)	361 (39.11)	1599 (27.41)	
No	4796 (70.99)	562 (60.89)	4234 (72.59)	
Drinking history (*n*, %)				< 0.001
Yes	1585 (23.46)	267 (28.93)	1318 (22.60)	
No	5171 (76.54)	656 (71.07)	4515 (77.40)	
LOS (day)	10.00 (7.00, 14.00)	12.00 (7.00, 21.00)	9.00 (7.00, 14.00)	< 0.001
*Diagnosis*				
Hypertension (*n*, %)				0.067
Yes	4350 (64.39)	569 (61.65)	3781 (64.82)	
No	2406 (35.61)	354 (38.35)	2052 (35.18)	
CHD (*n*, %)				< 0.001
Yes	2223 (32.90)	254 (27.52)	1969 (33.76)	
No	4533 (67.10)	669 (72.48)	3864 (66.24)	
CI (*n*, %)				0.001
Yes	1221 (18.07)	131 (14.19)	1090 (18.69)	
No	5535 (81.93)	792 (85.81)	4743 (81.31)	
Hyperlipidemia (*n*, %)				< 0.001
Yes	1026 (15.19)	83 (8.99)	943 (16.17)	
No	5730 (84.81)	840 (91.01)	4890 (83.83)	
*Laboratory measurements*				
AST (IU/L)	20.00 (16.00, 26.00)	20.34 (15.50, 28.00)	20.00 (16.00, 25.40)	0.091
ALT (IU/L)	18.00 (13.00, 26.00)	18.00 (12.89, 29.00)	18.00 (13.00, 26.00)	0.105
TGs (mmol/L)	1.42 (1.04, 2.04)	1.38 (1.00, 1.94)	1.43 (1.04, 2.06)	0.009
NLR	2.98 (2.08, 4.74)	3.39 (2.31, 6.07)	2.91 (2.06, 4.56)	< 0.001
LMR	3.88 (2.59, 5.48)	3.39 (2.13, 4.77)	3.97 (2.68, 5.57)	< 0.001
PLR	124.38 (91.01, 172.37)	135.79 (96.55, 199.74)	122.67 (90.29, 168.69)	< 0.001
NPAR	16.79 (14.63, 19.44)	18.07 (15.32, 22.12)	16.62 (14.55, 19.16)	< 0.001
CREA (*μ*mol/L)	72.00 (57.90, 92.10)	72.50 (59.15, 94.10)	72.00 (57.70, 91.70)	0.084
UA (*μ*mol/L)	326.25 (260.58, 399.00)	321.80 (257.35, 402.00)	326.90 (261.00, 398.80)	0.463
LDL-C (mmol/L)	2.49 (1.90, 3.14)	2.37 (1.87, 2.99)	2.51 (1.91, 3.17)	< 0.001
HDL-C (mmol/L)	1.12 (0.93, 1.35)	1.06 (0.88, 1.29)	1.13 (0.94, 1.36)	< 0.001
ALB (g/L)	40.60 (37.50, 43.60)	39.00 (34.45, 42.30)	40.80 (37.80, 43.70)	< 0.001
GFR (mL/min)	83.55 (63.03, 101.95)	85.87 (64.78, 102.97)	83.16 (62.91, 101.68)	0.27
*Vital signs*				
SBP (mmHg)	139.00 (126.00, 154.00)	136.00 (124.00, 150.00)	140.00 (127.00, 154.00)	0.001
DBP (mmHg)	78.00 (70.00, 85.00)	77.00 (70.00, 85.00)	78.00 (70.00, 85.00)	0.229
*Medication administration*				
Nutritional support drug use (*n*, %)				< 0.001
Yes	1708 (25.28)	510 (55.25)	1198 (20.54)	
No	5048 (74.72)	413 (44.75)	4635 (79.46)	
Antihypertensive drug use (*n*, %)				0.097
Yes	4369 (64.67)	574 (62.19)	3795 (65.06)	
No	2387 (35.33)	349 (37.81)	2038 (34.94)	
Antidiabetic drug use (*n*, %)				0.004
Yes	5079 (75.18)	658 (71.29)	4421 (75.79)	
No	1677 (24.82)	265 (28.71)	1412 (24.21)	
Statin drug use (*n*, %)				< 0.001
Yes	3405 (50.40)	323 (34.99)	3082 (52.84)	
No	3351 (49.60)	600 (65.01)	2751 (47.16)	
Antiplatelet and anticoagulant drug use (*n*, %)				< 0.001
Yes	4076 (60.33)	436 (47.24)	3640 (62.40)	
No	2680 (39.67)	487 (52.76)	2193 (37.60)	
Chinese patent medicine use (*n*, %)				< 0.001
Yes	5655 (83.70)	706 (76.49)	4949 (84.84)	
No	1101 (16.30)	217 (23.51)	884 (15.16)	
Analgesic drug use (*n*, %)				< 0.001
Yes	1579 (23.37)	444 (48.10)	1135 (19.46)	
No	5177 (76.63)	479 (51.90)	4698 (80.54)	
Antiosteoporotic drug use (*n*, %)				0.728
Yes	887 (13.13)	125 (13.54)	762 (13.06)	
No	5869 (86.87)	798 (86.46)	5071 (86.94)	
Antibiotic drug use (*n*, %)				< 0.001
Yes	2666 (39.46)	550 (59.59)	2116 (36.28)	
No	4090 (60.54)	373 (40.41)	3717 (63.72)	
Hemostatic agent drug use (*n*, %)				< 0.001
Yes	544 (8.05)	157 (17.01)	387 (6.63)	
No	6212 (91.95)	766 (82.99)	5446 (93.37)	
Psychotropic drug use (*n*, %)				< 0.001
Yes	1434 (21.23)	263 (28.49)	1171 (20.08)	
No	5322 (78.77)	660 (71.51)	4662 (79.92)	

Abbreviations: ACCI, age-adjusted Charlson comorbidity index; ALB, albumin; ALT, alanine aminotransferase; AST, aspartate aminotransferase; CHD, coronary heart disease; CI, cerebral infarction; CREA, creatinine; DBP, diastolic blood pressure; eGFR, estimated glomerular filtration rate; HDL-C, high-density lipoprotein cholesterol; LDL-C, low-density lipoprotein cholesterol; LMR, lymphocyte-to-monocyte ratio; LOS, length of stay; NLR, neutrophil-to-lymphocyte ratio; NPAR, neutrophil percentage-to-albumin ratio; PLR, platelet-to-lymphocyte ratio; PSH, past surgical history; SBP, systolic blood pressure; TGs, triglycerides; UA, uric acid; UEMI, urban employee medical insurance; URMI, urban resident medical insurance.

**Table 3:** Univariate analysis of factors associated with prolonged LOS.

**Variables**	**Training set (** **N** = 4729**)**	**Prolonged LOS (** **N** = 1344**)**	**Normal LOS (** **N** = 3385**)**	**p** ** value**
*Basic information*				
Age (year)	74.00 (69.00, 79.00)	74.00 (69.00, 80.00)	73.00 (69.00, 79.00)	0.190
Sex (*n*, %)				< 0.001
Female	2635 (55.72)	675 (50.22)	1960 (57.90)	
Male	2094 (44.28)	669 (49.78)	1425 (42.10)	
PSH (*n*, %)				0.004
Yes	3149 (66.59)	937 (69.72)	2212 (65.35)	
No	1580 (33.41)	407 (30.28)	1173 (34.65)	
Smoking history (*n*, %)				0.001
Yes	1348 (28.50)	429 (31.92)	919 (27.15)	
No	3381 (71.50)	915 (68.08)	2466 (72.85)	
Drinking history (*n*, %)				0.043
Yes	1086 (22.96)	335 (24.93)	751 (22.19)	
No	3643 (77.04)	1009 (75.07)	2634 (77.81)	
*Diagnosis*				
Hypertension (*n*, %)				0.008
Yes	3061 (64.73)	909 (67.63)	2152 (63.57)	
No	1668 (35.27)	435 (32.37)	1233 (36.43)	
CHD (*n*, %)				0.022
Yes	1578 (33.37)	482 (35.86)	1096 (32.38)	
No	3151 (66.63)	862 (64.14)	2289 (67.62)	
CI (*n*, %)				< 0.001
Yes	867 (18.33)	364 (27.08)	503 (14.86)	
No	3862 (81.67)	980 (72.92)	2882 (85.14)	
Hyperlipidemia (*n*, %)				0.196
Yes	712 (15.06)	188 (13.99)	524 (15.48)	
No	4017 (84.94)	1156 (86.01)	2861 (84.52)	
*Laboratory measurements*				
AST (IU/L)	20.00 (16.00, 26.00)	20.00 (16.00, 29.00)	19.80 (16.00, 25.00)	< 0.001
ALT (IU/L)	18.00 (12.80, 26.00)	18.11 (12.65, 29.03)	17.30 (12.89, 25.00)	0.001
TGs (mmol/L)	1.42 (1.04, 2.03)	1.38 (1.00, 1.94)	1.43 (1.06, 2.06)	0.002
NLR	2.99 (2.09, 4.74)	3.61 (2.37, 6.23)	2.81 (2.01, 4.25)	< 0.001
LMR	3.84 (2.61, 5.44)	3.27 (2.11, 4.76)	4.09 (2.81, 5.69)	< 0.001
PLR	125.26 (91.05, 172.09)	133.46 (95.11, 191.08)	122.58 (90.00, 164.86)	< 0.001
NPAR	16.84 (14.64, 19.45)	18.88 (15.67, 22.32)	16.35 (14.33, 18.45)	< 0.001
CREA (*μ*mol/L)	72.00 (57.90, 92.00)	75.60 (59.30, 98.53)	70.90 (57.40, 89.10)	< 0.001
UA (*μ*mol/L)	326.00 (260.80, 399.27)	314.50 (252.95, 397.05)	329.16 (263.66, 400.00)	0.010
LDL-C (mmol/L)	2.48 (1.90, 3.15)	2.36 (1.78, 2.99)	2.52 (1.95, 3.19)	< 0.001
HDL-C (mmol/L)	1.13 (0.94, 1.36)	1.08 (0.88, 1.33)	1.14 (0.95, 1.37)	< 0.001
ALB (g/L)	40.40 (37.40, 43.60)	37.90 (34.18, 41.71)	41.10 (38.50, 44.08)	< 0.001
GFR (mL/min)	83.67 (63.14, 102.32)	81.35 (58.97, 100.29)	84.54 (64.54, 103.22)	< 0.001
*Vital signs*				
SBP (mmHg)	139.00 (127.00, 154.00)	139.00 (126.00, 153.00)	139.00 (127.00, 154.00)	0.403
DBP (mmHg)	78.00 (70.00, 85.00)	78.00 (70.00, 85.00)	78.00 (70.00, 85.00)	0.344

Abbreviations: ALB, albumin; ALT, alanine aminotransferase; AST, aspartate aminotransferase; CHD, coronary heart disease; CI, cerebral infarction; CREA, creatinine; DBP, diastolic blood pressure; eGFR, estimated glomerular filtration rate; HDL-C, high-density lipoprotein cholesterol; LDL-C, low-density lipoprotein cholesterol; LMR, lymphocyte-to-monocyte ratio; NLR, neutrophil-to-lymphocyte ratio; NPAR, neutrophil percentage-to-albumin ratio; PLR, platelet-to-lymphocyte ratio; PSH, past surgical history; SBP, systolic blood pressure; TGs, triglycerides; UA, uric acid.

**Table 4:** Univariate analysis of factors associated with 30-day readmission.

**Variables**	**Training set (** **N** = 4729**)**	**30-day readmission (** **N** = 647**)**	**Non-30-day readmission (** **N** = 4082**)**	**p** ** value**
*Basic information*				
Age (year)	74.00 (69.00, 79.00)	73.00 (68.00, 78.00)	74.00 (69.00, 79.00)	0.009
Sex (*n*, %)				< 0.001
Female	2635 (55.72)	276 (42.66)	2359 (57.79)	
Male	2094 (44.28)	371 (57.34)	1723 (42.21)	
Insurance type (*n*, %)				0.047
Full self-pay	388 (8.20)	45 (6.96)	343 (8.40)	
UEMI	3242 (68.56)	456 (70.48)	2786 (68.25)	
URMI	871 (18.42)	127 (19.63)	744 (18.23)	
Other insurance	228 (4.82)	19 (2.94)	209 (5.12)	
ACCI score (*n*, %)				0.090
< 8	1238 (26.18)	187 (28.90)	1051 (25.75)	
≥ 8	3491 (73.82)	460 (71.10)	3031 (74.25)	
PSH (*n*, %)				0.917
Yes	3149 (66.59)	432 (66.77)	2717 (66.56)	
No	1580 (33.41)	215 (33.23)	1365 (33.44)	
Smoking history (*n*, %)				< 0.001
Yes	1348 (28.50)	245 (37.87)	1103 (27.02)	
No	3381 (71.50)	402 (62.13)	2979 (72.98)	
Drinking history (*n*, %)				0.001
Yes	1086 (22.96)	181 (27.98)	905 (22.17)	
No	3643 (77.04)	466 (72.02)	3177 (77.83)	
LOS (day)	9.00 (7.00, 14.00)	12.00 (7.00, 21.00)	9.00 (6.00, 14.00)	< 0.001
*Diagnosis*				
Hypertension (*n*, %)				0.080
Yes	3061 (64.73)	399 (61.67)	2662 (65.21)	
No	1668 (35.27)	248 (38.33)	1420 (34.79)	
CHD (*n*, %)				<0.001
Yes	1578 (33.37)	170 (26.28)	1408 (34.49)	
No	3151 (66.63)	477 (73.72)	2674 (65.51)	
CI (*n*, %)				< 0.001
Yes	867 (18.33)	85 (13.14)	782 (19.16)	
No	3862 (81.67)	562 (86.86)	3300 (80.84)	
Hyperlipidemia (*n*, %)				< 0.001
Yes	712 (15.06)	54 (8.35)	658 (16.12)	
No	4017 (84.94)	593 (91.65)	3424 (83.88)	
*Laboratory measurements*				
AST (IU/L)	20.00 (16.00, 26.00)	20.20 (15.55, 28.00)	19.99 (16.00, 25.49)	0.095
ALT (IU/L)	18.00 (12.80, 26.00)	18.50 (12.95, 29.00)	17.66 (12.80, 26.00)	0.057
TGs (mmol/L)	1.42 (1.04, 2.03)	1.39 (1.05, 1.95)	1.43 (1.04, 2.05)	0.156
NLR	2.99 (2.09, 4.74)	3.38 (2.24, 6.00)	2.92 (2.07, 4.56)	< 0.001
LMR	3.84 (2.61, 5.44)	3.40 (2.12, 4.77)	3.92 (2.66, 5.53)	< 0.001
PLR	125.26 (91.05, 172.09)	136.05 (96.45, 200.61)	123.48 (90.44, 167.70)	< 0.001
NPAR	16.84 (14.64, 19.45)	18.11 (15.29, 22.08)	16.70 (14.55, 19.18)	< 0.001
CREA (*μ*mol/L)	72.00 (57.90, 92.00)	72.40 (59.45, 91.50)	71.75 (57.80, 92.18)	0.206
UA (*μ*mol/L)	326.00 (260.80, 399.27)	318.60 (255.55, 398.25)	327.00 (261.90, 399.29)	0.110
LDL-C (mmol/L)	2.48 (1.90, 3.15)	2.36 (1.86, 2.99)	2.49 (1.91, 3.17)	0.002
HDL-C (mmol/L)	1.13 (0.94, 1.36)	1.07 (0.89, 1.29)	1.13 (0.94, 1.36)	< 0.001
ALB (g/L)	40.40 (37.40, 43.60)	38.94 (34.30, 42.30)	40.67 (37.70, 43.70)	< 0.001
GFR (mL/min)	83.67 (63.14, 102.32)	86.42 (66.81, 102.97)	83.30 (62.89, 102.09)	0.200
*Vital signs*				
SBP (mmHg)	139.00 (127.00, 154.00)	136.00 (124.00, 150.00)	140.00 (127.00, 154.00)	0.002
DBP (mmHg)	78.00 (70.00, 85.00)	77.00 (70.00, 84.00)	78.00 (70.00, 85.00)	0.049
*Medication administration*				
Nutritional support drug use (*n*, %)				< 0.001
Yes	1219 (25.78)	363 (56.11)	856 (20.97)	
No	3510 (74.22)	284 (43.89)	3226 (79.03)	
Antihypertensive drug use (*n*, %)				0.026
Yes	3049 (64.47)	392 (60.59)	2657 (65.09)	
No	1680 (35.53)	255 (39.41)	1425 (34.91)	
Antidiabetic drug use (*n*, %)				0.167
Yes	3546 (74.98)	471 (72.80)	3075 (75.33)	
No	1183 (25.02)	176 (27.20)	1007 (24.67)	
Statin drug use (*n*, %)				< 0.001
Yes	2357 (49.84)	224 (34.62)	2133 (52.25)	
No	2372 (50.16)	423 (65.38)	1949 (47.75)	
Antiplatelet and anticoagulant drug use (*n*, %)				< 0.001
Yes	2831 (59.86)	302 (46.68)	2529 (61.95)	
No	1898 (40.14)	345 (53.32)	1553 (38.05)	
Chinese patent medicine use (*n*, %)				< 0.001
Yes	3969 (83.93)	493 (76.20)	3476 (85.15)	
No	760 (16.07)	154 (23.80)	606 (14.85)	
Analgesic drug use (*n*, %)				< 0.001
Yes	1134 (23.98)	307 (47.45)	827 (20.26)	
No	3595 (76.02)	340 (52.55)	3255 (79.74)	
Antiosteoporotic drug use (*n*, %)				0.819
Yes	620 (13.11)	83 (12.83)	537 (13.16)	
No	4109 (86.89)	564 (87.17)	3545 (86.84)	
Antibiotic drug use (*n*, %)				< 0.001
Yes	1903 (40.24)	386 (59.66)	1517 (37.16)	
No	2826 (59.76)	261 (40.34)	2565 (62.84)	
Hemostatic agent drug use (*n*, %)				< 0.001
Yes	395 (8.35)	112 (17.31)	283 (6.93)	
No	4334 (91.65)	535 (82.69)	3799 (93.07)	
Psychotropic drug use (*n*, %)				< 0.001
Yes	1021 (21.59)	185 (28.59)	836 (20.48)	
No	3708 (78.41)	462 (71.41)	3246 (79.52)	

Abbreviations: ACCI, age-adjusted Charlson comorbidity index; ALB, albumin; ALT, alanine aminotransferase; AST, aspartate aminotransferase; CHD, coronary heart disease; CI, cerebral infarction; CREA, creatinine; DBP, diastolic blood pressure; eGFR, estimated glomerular filtration rate; HDL-C, high-density lipoprotein cholesterol; LDL-C, low-density lipoprotein cholesterol; LMR, lymphocyte-to-monocyte ratio; LOS, length of stay; NLR, neutrophil-to-lymphocyte ratio; NPAR, neutrophil percentage-to-albumin ratio; PLR, platelet-to-lymphocyte ratio; PSH, past surgical history; SBP, systolic blood pressure; TGs, triglycerides; UA, uric acid; UEMI, urban employee medical insurance; URMI, urban resident medical insurance.

**Table 5:** Detailed performance metrics of the prolonged LOS model for the four sets.

**Models**	**AUC**	**Sensitivity**	**Specificity**	**PPV**	**NPV**	**Youden's index**
	**(95% CI)**	**(95% CI)**	**(95% CI)**	**(95% CI)**	**(95% CI)**	**(95% CI)**
Training set	0.720	0.592	0.780	0.517	0.828	0.372
	0.703–0.737	0.566–0.619	0.766–0.794	0.492–0.542	0.815–0.841	0.332–0.413
Internal validation set	0.715	0.615	0.767	0.500	0.840	0.382
	0.688–0.742	0.574–0.655	0.745–0.788	0.463–0.537	0.820–0.859	0.319–0.443
External Validation Set I	0.736	0.578	0.848	0.454	0.902	0.426
	0.696–0.777	0.514–0.643	0.826–0.870	0.396–0.512	0.883–0.921	0.340–0.513
External Validation Set II	0.703	0.544	0.849	0.348	0.926	0.393
	0.645–0.761	0.447–0.640	0.823–0.876	0.274–0.421	0.906–0.947	0.270–0.516

Abbreviations: AUROC, area under the receiver operating characteristic curve; CI, confidence interval; NPV, negative predictive value; PPV, positive predictive value.

**Table 6:** Detailed performance metrics of the 30-day readmission model for the four datasets.

**Models**	**AUC**	**Sensitivity**	**Specificity**	**PPV**	**NPV**	**Youden's index**
	**(95% CI)**	**(95% CI)**	**(95% CI)**	**(95% CI)**	**(95% CI)**	**(95% CI)**
Training set	0.766	0.675	0.787	0.335	0.939	0.462
	0.745–0.787	0.639–0.712	0.775–0.800	0.309–0.360	0.931–0.947	0.414–0.512
Internal validation set	0.769	0.612	0.847	0.387	0.933	0.459
	0.736–0.801	0.555–0.670	0.830–0.864	0.341–0.432	0.920–0.945	0.385–0.534
External Validation Set I	0.633	0.554	0.682	0.313	0.854	0.236
	0.594–0.673	0.494–0.615	0.652–0.711	0.270–0.355	0.829–0.879	0.146–0.326
External Validation Set II	0.671	0.548	0.741	0.218	0.926	0.289
	0.613–0.729	0.447–0.650	0.709–0.773	0.165–0.271	0.906–0.947	0.156–0.423

Abbreviations: AUROC, area under the receiver operating characteristic curve; CI, confidence interval; NPV, negative predictive value; PPV, positive predictive value.

**Table 7:** The AUROCs in the prolonged LOS and 30-day readmission models of different age subgroups.

**Models**	**Group 1 (< 75 years)**	**Group 2 (75~84 years)**	**Group 3 (> 84 years)**
*Prolonged LOS*			
Training set	0.706 (0.682–0.731)	0.738 (0.712–0.764)	0.714 (0.655–0.774)
Internal validation set	0.718 (0.682–0.755)	0.702 (0.657–0.746)	0.725 (0.643–0.806)
External Validation Set I	0.741 (0.689–0.792)	0.716 (0.640–0.792)	0.785 (0.658–0.912)
External Validation Set II	0.726 (0.644–0.808)	0.672 (0.577–0.768)	0.645 (0.478–0.812)
*30-day readmission*			
Training set	0.777 (0.750–0.804)	0.736 (0.697–0.776)	0.814 (0.751–0.877)
Internal validation set	0.776 (0.735–0.818)	0.745 (0.687–0.803)	0.804 (0.700–0.908)
External Validation Set I	0.623 (0.571–0.674)	0.658 (0.592–0.723)	0.611 (0.442–0.780)
External Validation Set II	0.631 (0.535–0.727)	0.690 (0.611–0.769)	0.712 (0.516–0.918)

*Note:* Data in parentheses are 95% confidence intervals.

Abbreviation: LOS, length of stay.

## Data Availability

The datasets used for this study are available on request to the corresponding author.

## Supporting Information

Supporting Information Additional supporting information can be found online in the Supporting Information section. This section includes a comparison of the datasets before and after multiple imputation; predictive performance metrics for prolonged LOS and 30-day readmission models, including sensitivity, specificity, NPV, PPV, and brier score; model evaluation plots, such as the ROC curve, calibration curve, decision curve, and clinical impact curve; and subgroup analysis results stratified by age.

## References

[1] GBD 2021 Diabetes Collaborators (2023). Global, Regional, and National Burden of Diabetes From 1990 to 2021, With Projections of Prevalence to 2050: A Systematic Analysis for the Global Burden of Disease Study 2021. *Lancet*.

[2] Gregg E. W., Pratt A., Owens A. (2024). The Burden of Diabetes-Associated Multiple Long-Term Conditions on Years of Life Spent and Lost. *Nature Medicine*.

[3] Sun H., Saeedi P., Karuranga S. (2022). IDF Diabetes Atlas: Global, Regional and Country-Level Diabetes Prevalence Estimates for 2021 and Projections for 2045. *Diabetes Research and Clinical Practice*.

[4] Tu W.-J., Xue Y., Nie D. (2022). The Prevalence and Treatment of Diabetes in China From 2013 to 2018. *Journal of the American Medical Association*.

[5] NCD Risk Factor Collaboration (NCD-RisC) (2016). Worldwide Trends in Diabetes Since 1980: A Pooled Analysis of 751 Population-Based Studies With 4·4 Million Participants. *Lancet*.

[6] Liu J., Liu M., Chai Z. (2023). Projected Rapid Growth in Diabetes Disease Burden and Economic Burden in China: A Spatio-Temporal Study From 2020 to 2030. *Lancet Regional Health-Western Pacific*.

[7] Tan J., Zhang Z., He Y. (2023). A Novel Model for Predicting Prolonged Stay of Patients With Type-2 Diabetes Mellitus: A 13-Year (2010-2022) Multicenter Retrospective Case-Control Study. *Journal of Translational Medicine*.

[8] Caughey G. E., Pratt N. L., Barratt J. D., Shakib S., Kemp-Casey A. R., Roughead E. E. (2017). Understanding 30-Day Re-Admission After Hospitalisation of Older Patients for Diabetes: Identifying Those at Greatest Risk. *Medical Journal of Australia*.

[9] AlBekairy A., AbuRuz S., Alsabani B. (2018). Exploring Factors Associated With Depression and Anxiety Among Hospitalized Patients With Type 2 Diabetes Mellitus. *Medical Principles and Practice*.

[10] Kaye K. S., Marchaim D., Chen T. Y. (2014). Effect of Nosocomial Bloodstream Infections on Mortality, Length of Stay, and Hospital Costs in Older Adults. *Journal of the American Geriatrics Society*.

[11] Maksimowicz-McKinnon K., Zhou J., Hudy J., Hegab S., McKinnon J. E. (2021). Subclinical CMV Viremia Is Associated With Increased Nosocomial Infections and Prolonged Hospitalization in Patients With Systemic Autoimmune Diseases. *Journal of Clinical Virology*.

[12] Wolkewitz M., Zortel M., Palomar-Martinez M., Alvarez-Lerma F., Olaechea-Astigarraga P., Schumacher M. (2017). Landmark Prediction of Nosocomial Infection Risk to Disentangle Short- and Long-Stay Patients. *The Journal of Hospital Infection*.

[13] Barsasella D., Gupta S., Malwade S. (2021). Predicting Length of Stay and Mortality Among Hospitalized Patients With Type 2 Diabetes Mellitus and Hypertension. *International Journal of Medical Informatics*.

[14] Stefan M. S., Pekow P. S., Nsa W. (2013). Hospital Performance Measures and 30-Day Readmission Rates. *Journal of General Internal Medicine*.

[15] Shang Y., Jiang K., Wang L. (2021). The 30-Days Hospital Readmission Risk in Diabetic Patients: Predictive Modeling With Machine Learning Classifiers. *BMC Medical Informatics and Decision Making*.

[16] Ossai C. I., Wickramasinghe N. (2022). A Hybrid Approach for Risk Stratification and Predictive Modelling of 30-Days Unplanned Readmission of Comorbid Patients With Diabetes. *Journal of Diabetes and its Complications*.

[17] Bansal V., Mottalib A., Pawar T. K. (2018). Inpatient Diabetes Management by Specialized Diabetes Team Versus Primary Service Team in Non-Critical Care Units: Impact on 30-Day Readmission Rate and Hospital Cost. *BMJ Open Diabetes Research & Care*.

[18] Jacobs C. C., Jaber J. F., Ladna M. (2021). Factors Associated With Inpatient Endoscopy Delay and Its Impact on Hospital Length-of-Stay and 30-Day Readmission. *Clinical Gastroenterology and Hepatology*.

[19] Klemt C., Tirumala V., Barghi A., Cohen-Levy W. B., Robinson M. G., Kwon Y.-M. (2022). Artificial Intelligence Algorithms Accurately Predict Prolonged Length of Stay Following Revision Total Knee Arthroplasty. *Knee Surgery, Sports Traumatology, Arthroscopy*.

[20] Hsu T. Y., Wang P. M., Chuang P. C. (2022). The Impact of Do-Not-Resuscitate Order in the Emergency Department on Respiratory Failure After ICU Admission. *Healthcare*.

[21] Wei D., Sun Y., Chen R., Meng Y., Wu W. (2023). Age-Adjusted Charlson Comorbidity Index and In-Hospital Mortality in Critically Ill Patients With Cardiogenic Shock: A Retrospective Cohort Study. *Experimental and Therapeutic Medicine*.

[22] Matsushita K., Ballew S. H., Coresh J. (2017). Measures of Chronic Kidney Disease and Risk of Incident Peripheral Artery Disease: A Collaborative Meta-Analysis of Individual Participant Data. *The Lancet Diabetes and Endocrinology*.

[23] Erdogan A., Can F. E., Gönüllü H. (2021). Evaluation of the Prognostic Role of NLR, LMR, PLR and LCR Ratio in COVID-19 Patients. *Journal of Medical Virology*.

[24] Gambardella C., Mongardini F. M., Paolicelli M. (2023). Role of Inflammatory Biomarkers (NLR, LMR, PLR) in the Prognostication of Malignancy in Indeterminate Thyroid Nodules. *International Journal of Molecular Sciences*.

[25] Cai J., Li M., Wang W., Luo R., Zhang Z., Liu H. (2023). The Relationship Between the Neutrophil Percentage-to-Albumin Ratio and Rates of 28-Day Mortality in Atrial Fibrillation Patients 80 Years of Age or Older. *Journal of Inflammation Research*.

[26] Collins G. S., Reitsma J. B., Altman D. G., Moons K. G. M. (2015). Transparent Reporting of a Multivariable Prediction Model for Individual Prognosis or Diagnosis (TRIPOD): The TRIPOD Statement. *Annals of Internal Medicine*.

[27] Soh J. G. S., Mukhopadhyay A., Mohankumar B., Quek S. C., Tai B. C. (2022). Predicting and Validating 30-Day Hospital Readmission in Adults With Diabetes Whose Index Admission Is Diabetes-Related. *The Journal of Clinical Endocrinology and Metabolism*.

[28] Collins J., Abbass I. M., Harvey R. (2017). Predictors of All-Cause 30 Day Readmission Among Medicare Patients With Type 2 Diabetes. *Current Medical Research and Opinion*.

[29] Sherman E., Alejo D., Wood-Doughty Z. (2022). Leveraging Machine Learning to Predict 30-Day Hospital Readmission After Cardiac Surgery. *The Annals of Thoracic Surgery*.

[30] Larsson S. C., Scott R. A., Traylor M. (2017). Type 2 Diabetes, Glucose, Insulin, BMI, and Ischemic Stroke Subtypes. *Neurology*.

[31] Chen R., Ovbiagele B., Feng W. (2016). Diabetes and Stroke: Epidemiology, Pathophysiology, Pharmaceuticals and Outcomes. *The American Journal of the Medical Sciences*.

[32] Yang R., Pedersen N. L., Bao C. (2019). Type 2 Diabetes in Midlife and Risk of Cerebrovascular Disease in Late Life: A Prospective Nested Case-Control Study in a Nationwide Swedish Twin Cohort. *Diabetologia*.

[33] Shah A. D., Langenberg C., Rapsomaniki E. (2015). Type 2 Diabetes and Incidence of Cardiovascular Diseases: A Cohort Study in 1·9 Million People. *The Lancet Diabetes and Endocrinology*.

[34] Tang Y., Hou H., Li L. (2022). Neutrophil Percentage-to-Albumin Ratio: A Good Parameter for the Evaluation of the Severity of Anti-NMDAR Encephalitis at Admission and Prediction of Short-Term Prognosis. *Frontiers in Immunology*.

[35] Ko C. A., Fang K. H., Tsai M. S. (2022). Prognostic Value of Neutrophil Percentage-to-Albumin Ratio in Patients With Oral Cavity Cancer. *Cancers*.

[36] He H. M., Lin X., Luo M. (2021). Predictive Value of Neutrophil Percentage-to-Albumin Ratio for Contrast-Associated Acute Kidney Injury in Patients Without Chronic Kidney Disease Undergoing Elective Percutaneous Coronary Intervention. *European Heart Journal*.

[37] Sobhani S., Aryan R., AkbariRad M. (2021). The Association Between Anthropometry Indices and Serum Concentrations of Gamma-Glutamyl Transferase, Alkaline Phosphatase, Alanine Aminotransferase, and Aspartate Aminotransferase. *BioMed Research International*.

[38] Toh M. R., Teo V., Kwan Y. H., Raaj S., Tan S.-Y. D., Tan J. Z. Y. (2014). Association Between Number of Doses per Day, Number of Medications and Patient's Non-Compliance, and Frequency of Readmissions in a Multi-Ethnic Asian Population. *Preventive Medical Reports*.

[39] Capoccia K., Odegard P. S., Letassy N. (2016). Medication Adherence With Diabetes Medication. *The Diabetes Educator*.

[40] Fadini G. P., Cosentino F. (2018). Diabetes and Ischaemic Stroke: A Deadly Association. *European Heart Journal*.

[41] El Naamani K., Hunt A., Jain P. (2023). The Rate and Predictors of 30-Day Readmission in Patients Treated for Unruptured Cerebral Aneurysms: A Large Single-Center Study. *Neurosurgery*.

[42] Regassa L. D., Tola A. (2021). Magnitude and Predictors of Hospital Admission, Readmission, and Length of Stay Among Patients With Type 2 Diabetes at Public Hospitals of Eastern Ethiopia: A Retrospective Cohort Study. *BMC Endocrine Disorders*.

[43] Chakraborty A., Pearson O., Schwartzkopff K. M. (2021). The Effectiveness of In-Hospital Interventions on Reducing Hospital Length of Stay and Readmission of Patients With Type 2 Diabetes Mellitus: A Systematic Review. *Diabetes Research and Clinical Practice*.

[44] Anraku M., Chuang V. T. G., Maruyama T., Otagiri M. (2013). Redox Properties of Serum Albumin. *Biochimica et Biophysica Acta*.

[45] Paar M., Rossmann C., Nusshold C. (2017). Anticoagulant Action of Low, Physiologic, and High Albumin Levels in Whole Blood. *PLoS One*.

[46] Wada H., Dohi T., Miyauchi K. (2017). Impact of Serum Albumin Levels on Long-Term Outcomes in Patients Undergoing Percutaneous Coronary Intervention. *Heart and Vessels*.

[47] Yang C., Lu J., Shen F., Xie H., Cui H., Xu R. (2024). Serum Albumin Level Is Associated With Mortality and Hospital Stays: A Real-World Data Analysis. *Clinical Nutrition ESPEN*.

[48] Tang W., Yao W., Wang W., Ding W., Ni X., He R. (2024). Association Between Admission Albumin Levels and 30-Day Readmission After Hip Fracture Surgery in Geriatric Patients: A Propensity Score-Matched Study. *BMC Musculoskeletal Disorders*.

[49] Tang M., Zhao Y., Xiao J. (2024). Development and Validation of a Predictive Model for Prolonged Length of Stay in Elderly Type 2 Diabetes Mellitus Patients Combined With Cerebral Infarction. *Frontiers in Neurology*.

[50] Wiedermann C. J. (2021). Hypoalbuminemia as Surrogate and Culprit of Infections. *International Journal of Molecular Sciences*.

[51] Cheng T., Wang X., Han Y., Hao J., Hu H., Hao L. (2023). The Level of Serum Albumin Is Associated With Renal Prognosis and Renal Function Decline in Patients With Chronic Kidney Disease. *BMC Nephrology*.

[52] Kato T., Yaku H., Morimoto T. (2020). Association of an Increase in Serum Albumin Levels With Positive 1-Year Outcomes in Acute Decompensated Heart Failure: A Cohort Study. *PLoS One*.

[53] Cai Y. W., Zhang H. F., Gao J. W. (2023). Serum Albumin and Risk of Incident Diabetes and Diabetic Microvascular Complications in the UK Biobank Cohort. *Diabetes & Metabolism*.

[54] Mayne K. J., Sardell R. J., Staplin N. (2024). Frailty, Multimorbidity, and Polypharmacy: Exploratory Analyses of the Effects of Empagliflozin from the EMPA-KIDNEY Trial. *Clinical Journal of the American Society of Nephrology*.

[55] Mone P., Guerra G., Lombardi A. (2024). Effects of SGLT2 Inhibition via Empagliflozin on Cognitive and Physical Impairment in Frail Diabetic Elders With Chronic Kidney Disease. *Pharmacological Research*.

[56] Santulli G., Varzideh F., Forzano I. (2023). Functional and Clinical Importance of SGLT2-Inhibitors in Frailty: From the Kidney to the Heart. *Hypertension*.

[57] Pollack R., Cahn A. (2022). SGLT2 Inhibitors and Safety in Older Patients. *Heart Failure Clinics*.

[58] Malik M. E., Butt J. H., Strange J. E. (2023). Initiation of SGLT2 Inhibitors and GLP-1 Receptor Agonists According to Level of Frailty in People With Type 2 Diabetes and Cardiovascular Disease in Denmark: A Cross-Sectional, Nationwide Study. *Lancet Healthy Longevity*.

[59] Aldafas R., Crabtree T., Alkharaiji M., Vinogradova Y., Idris I. (2024). Sodium-Glucose Cotransporter-2 Inhibitors (SGLT2) in Frail or Older People With Type 2 Diabetes and Heart Failure: A Systematic Review and Meta-Analysis. *Age and Ageing*.

[60] Mone P., Ciccarelli M., Jankauskas S. S. (2024). SGLT2 Inhibitors and GLP-1 Receptor Agonists: Which Is the Best Anti-Frailty Drug?. *Lancet Healthy Longevity*.

