# Supplementary material for: Derivation and Validation of Prediction Models for Prolonged Length of Stay and 30-Day Readmission in Elderly Patients With Type 2 Diabetes Mellitus: A Multicenter Study
Source: J Diabetes Res. 2025 May 12;2025:3148242. doi: 10.1155/jdr/3148242 (PMC12088840; doi:10.1155/jdr/3148242)

**Supplementary information**

**Table S1.** TRIPOD Checklist: Development and Validation of Prediction Model.

| **Section/Topic** | **Item** |  | **Checklist item** | **Page** |
| --- | --- | --- | --- | --- |
| **Title and abstract** | | | | |
| Title | 1 | D;V | Identify the study as developing and/or validating a multivariable prediction model, the target population, and the outcome to be predicted. | 1 |
| Abstract | 2 | D;V | Provide a summary of objectives, study design, setting, participants, sample size, predictors, outcome, statistical analysis, results, and conclusions. | 2 |
| **Introduction** | | | | |
| Background and objectives | 3a | D;V | Explain the medical context (including whether diagnostic or prognostic) and rationale for developing or validating the multivariable prediction model, including references to existing models. | 3-4 |
|  | 3b | D;V | Specify the objectives, including whether the study describes the development or validation of the model or both. | 5 |
| **Methods** | | | | |
| Source of data | 4a | D;V | Describe the study design or source of data (e.g., randomized trial, cohort, or registry data), separately for the development and validation datasets, if applicable. | 4 |
|  | 4b | D;V | Specify the key study dates, including start of accrual; end of accrual; and, if applicable, end of follow-up. | 4 |
| Participants | 5a | D;V | Specify key elements of the study setting (e.g., primary care, secondary care, general population) including number and location of centres. | 4-5 |
|  | 5b | D;V | Describe eligibility criteria for participants. | 4 |
|  | 5c | D;V | Give details of treatments received, if relevant. | NA |
| Outcome | 6a | D;V | Clearly define the outcome that is predicted by the prediction model, including how and when assessed. | 4-5 |
|  | 6b | D;V | Report any actions to blind assessment of the outcome to be predicted. | NA |
| Predictors | 7a | D;V | Clearly define all predictors used in developing or validating the multivariable prediction model, including how and when they were measured. | 4-5 |
|  | 7b | D;V | Report any actions to blind assessment of predictors for the outcome and other predictors. | NA |
| Sample size | 8 | D;V | Explain how the study size was arrived at. | 6 |
| Missing data | 9 | D;V | Describe how missing data were handled (e.g., complete-case analysis, single imputation, multiple imputation) with details of any imputation method. | 5 |
| Statistical analysis methods | 10a | D | Describe how predictors were handled in the analyses. | 5 |
|  | 10b | D | Specify type of model, all model-building procedures (including any predictorselection), and method for internal validation. | 5 |
|  | 10c | V | For validation, describe how the predictions were calculated. | 7 |
|  | 10d | D;V | Specify all measures used to assess model performance and, if relevant, to compare multiple models. | 5, 7-8 |
|  | 10e | V | Describe any model updating (e.g., recalibration) arising from the validation, if done. | NA |
| Risk groups | 11 | D;V | Provide details on how risk groups were created, if done. | NA |
| Development vs. validation | 12 | V | For validation, identify any differences from the development data in setting, eligibility criteria, outcome, and predictors. | 6-8, Table 3 & 4 |
| **Results** | | | | |
| Participants | 13a | D;V | Describe the flow of participants through the study, including the number of participants with and without the outcome and, if applicable, a summary of the follow-up time. A diagram maybe helpful. | Table 1 & 2 |
|  | 13b | D;V | Describe the characteristics of the participants (basic demographics, clinical features, available predictors), including the number of participants with missing data for predictors and outcome. | 6-8, Table 1 & 2 |
|  | 13c | V | For validation, show a comparison with the development data of the distribution of important variables (demographics, predictors and outcome). | 7-8, Table 3 & 4 |
| Model development | 14a | D | Specify the number of participants and outcome events in each analysis. | 7-8, Table 3 & 4 |
|  | 14b | D | If done, report the unadjusted association between each candidate predictor and outcome. | 6, Table 1 & 2 |
| Model specification | 15a | D | Present the full prediction model to allow predictions for individuals (i.e., all regression coefficients, and model interceptor baseline survival at a given time point). | 7-8, Figure 3 & 5 |
|  | 15b | D | Explain how to use the prediction model. | 7-8, Figure 5 & 7 |
| Model performance | 16 | D;V | Report performance measures (with CIs) for the prediction model. | Table 5-7 |
| Model-updating | 17 | V | If done, report the results from any model updating (i.e., model specification, model performance). | NA |
| **Discussion** | | | | |
| Limitations | 18 | D;V | Discuss any limitations of the study (such as nonrepresentative sample, few events per predictor, missing data). | 10 |
| Interpretation | 19a | V | For validation, discuss the results with reference to performance in the development data, and any other validation data. | 8-9 |
|  | 19b | D;V | Give an overall interpretation of the results, considering objectives, limitations, results from similar studies, and other relevant evidence. | 8-10 |
| Implications | 20 | D;V | Discuss the potential clinical use of the model and implications for future research. | 8-10 |
| **Other information** | | | | |
| Supplementary information | 21 | D;V | Provide information about the availability of supplementary resources, such as study protocol, Web calculator, and data sets. | 7-8, dynamic web-based calculator |
| Funding | 22 | D;V | Give the source of funding and the role of the funders for the present study. | 11 |

*Items relevant only to the development of a prediction model are denoted by D, items relating

solely to a validation of a prediction model are denoted by V, and items relating to both are denoted

D;V. We recommend using the TRIPOD Checklist in conjunction with the TRIPOD Explanation and Elaboration document.

**Table S2.** Comparison of continuous variables in the training and internal validation sets before and after multiple imputation.

| **Missing variables** | **Before interpolation** | **After interpolation** | ***P* values** |
| --- | --- | --- | --- |
| SBP (mmHg) | 140.00(126.00,154.00) | 139.00(126.00,154.00) | 0.717 |
| DBP (mmHg) | 78.00(70.00,85.00) | 78.00(70.00,85.00) | 0.704 |
| AST (IU/L) | 20.00(16.00,26.00) | 20.00(16.00,26.00) | 0.158 |
| ALT (IU/L) | 18.00(13.00,26.08) | 18.00(13.00,26.00) | 0.482 |
| TGs (mmol/l) | 1.44(1.04,2.06) | 1.42(1.04,2.04) | 0.421 |
| NLR | 3.03(2.10,4.90) | 2.98(2.08,4.74) | 0.131 |
| LMR | 3.86(2.61,5.47) | 3.88(2.59,5.48) | 0.851 |
| PLR | 125.82(90.98,175.00) | 124.38(91.01,172.37) | 0.364 |
| NPAR | 16.84(14.66,19.61) | 16.79(14.63,19.44) | 0.249 |
| CREA (umol/l) | 72.25(58.20,92.73) | 72.00(57.90,92.10) | 0.415 |
| UA (umol/l) | 327.04(261.90,399.18) | 326.25(260.58,399.00) | 0.701 |
| LDL-C (mmol/l) | 2.50(1.91,3.17) | 2.49(1.90,3.14) | 0.394 |
| HDL-C (mmol/l) | 1.12(0.94,1.35) | 1.12(0.93,1.35) | 0.810 |
| ALB (g/L) | 40.60(37.40,43.60) | 40.60(37.50,43.60) | 0.793 |
| GFR (mL/min) | 83.51(62.91,101.74) | 83.55(63.03,101.95) | 0.821 |

*SBP: systolic blood pressure; DBP: diastolic blood pressure; AST: aspartate aminotransferase; ALT: alanine aminotransferase; TGs: triglycerides; CREA: creatinine; UA: uric acid; LDL-C: low-density lipoprotein cholesterol; HDL-C: high-density lipoprotein cholesterol; ALB: albumin; eGFR: estimated glomerular filtration rate; NLR: neutrophil-to-lymphocyte ratio; PLR: platelet-to-lymphocyte ratio; LMR: lymphocyte-to-monocyte ratio; NPAR: neutrophil percentage-to-albumin ratio.*

**Table S3.** The AUROC values for different numbers of predictor variables.

| **Number of Predictors** | **Ensemble Predictors** | **AUROC (95%CI)** | ***P* value** |
| --- | --- | --- | --- |
| Prolonged LOS |  |  |  |
| 1 | NPAR | 0.675 (0.657-0.693) |  |
| 2 | NPAR+ALB | 0.691 (0.673-0.709) |  |
| 3 | NPAR+ALB+CI | 0.713 (0.696-0.730) |  |
| 4 | NPAR+ALB+CI+AST | 0.720 (0.703-0.737) |  |
| 5 | NPAR+ALB+CI+AST+sex | 0.722 (0.705-0.739) | 0.131 |
| 30-day readmission |  |  |  |
| 1 | nutritional support drug use | 0.676 (0.656-0.696) |  |
| 2 | nutritional support drug use+analgesics drug use | 0.728 (0.707-0.748) |  |
| 3 | nutritional support drug use+analgesics drug use+LOS | 0.730 (0.707-0.753) |  |
| 4 | nutritional support drug use+analgesics drug use+LOS+ALB | 0.738 (0.715-0.762) |  |
| 5 | nutritional support drug use+analgesics drug use+LOS+ALB+sex | 0.753 (0.730-0.775) |  |
| 6 | nutritional support drug use+analgesics drug use+LOS+ALB+sex+antibiotics drug use | 0.755 (0.733-0.778) |  |
| 7 | nutritional support drug use+analgesics drug use+LOS+ALB+sex+antibiotics drug use+antiplatelet and anticoagulant drug use | 0.766 (0.745-0.787) |  |
| 8 | nutritional support drug use+analgesics drug use+LOS+ALB+sex+antibiotics drug use+antiplatelet and anticoagulant drug use+statin drug use | 0.767 (0.746-0.788) | 0.479 |

*NPAR: neutrophil percentage-to-albumin ratio; ALB: albumin; CI: cerebral infarction; AST: aspartate aminotransferase; LOS: length of stay.*

**Table S4.** The sensitivity in prolonged LOS and 30-day readmission models of different age subgroups.

| **Models** | **Group1 (<75 years)** | **Group2 (75~84 years)** | **Group3 (>84 years)** |
| --- | --- | --- | --- |
| **Prolonged LOS** |  |  |  |
| Training set | 0.569 (0.533-0.605) | 0.673 (0.632-0.713) | 0.713 (0.628-0.798) |
| Internal validation set | 0.585 (0.529-0.631) | 0.632 (0.566-0.697) | 0.780 (0.665-0.895) |
| External validation set Ⅰ | 0.538 (0.453-0.624) | 0.630 (0.519-0.741) | 0.850 (0.694-1.000) |
| External validation set Ⅱ | 0.500 (0.352-0.648) | 0.553 (0.411-0.695) | 0.833 (0.622-1.000) |
| **30-day readmission** |  |  |  |
| Training set | 0.668 (0.621-0.716) | 0.644 (0.580-0.707) | 0.833 (0.728-0.939) |
| Internal validation set | 0.655 (0.582-0.727) | 0.551 (0.447-0.654) | 0.727 (0.541-0.913) |
| External validation set Ⅰ | 0.525 (0.448-0.602) | 0.691 (0.591-0.792) | 0.667 (0.428-0.905) |
| External validation set Ⅱ | 0.459 (0.299-0.620) | 0.640 (0.507-0.773) | 0.833 (0.535-1.000) |

*Data in parentheses are 95% confidence intervals. LOS: length of stay.*

**Table S5.** The specificity in prolonged LOS and 30-day readmission models of different age subgroups.

| **Models** | **Group1 (<75 years)** | **Group2 (75~84 years)** | **Group3 (>84 years)** |
| --- | --- | --- | --- |
| **Prolonged LOS** |  |  |  |
| Training set | 0.799 (0.780-0.817) | 0.718 (0.694-0.743) | 0.676 (0.618-0.734) |
| Internal validation set | 0.799 (0.771-0.826) | 0.742 (0.705-0.779) | 0.658 (0.569-0.746) |
| External validation set Ⅰ | 0.883 (0.858-0.908) | 0.777 (0.733-0.822) | 0.705 (0.590-0.819) |
| External validation set Ⅱ | 0.876 (0.843-0.909) | 0.844 (0.801-0.887) | 0.500 (0.349-0.651) |
| **30-day readmission** |  |  |  |
| Training set | 0.806 (0.789-0.822) | 0.787 (0.766-0.807) | 0.756 (0.708-0.804) |
| Internal validation set | 0.815 (0.791-0.840) | 0.883 (0.859-0.908) | 0.827 (0.765-0.890) |
| External validation set Ⅰ | 0.711 (0.674-0.747) | 0.608 (0.555-0.661) | 0.652 (0.537-0.766) |
| External validation set Ⅱ | 0.751 (0.708-0.794) | 0.685 (0.630-0.740) | 0.750 (0.627-0.873) |

*Data in parentheses are 95% confidence intervals. LOS: length of stay.*

**Table S6.** The NPVs in prolonged LOS and 30-day readmission models of different age subgroups.

| **Models** | **Group1 (<75 years)** | **Group2 (75~84 years)** | **Group3 (>84 years)** |
| --- | --- | --- | --- |
| **Prolonged LOS** |  |  |  |
| Training set | 0.828 (0.810-0.845) | 0.844 (0.822-0.866) | 0.843 (0.793-0.894) |
| Internal validation set | 0.841 (0.815-0.866) | 0.839 (0.806-0.872) | 0.869 (0.797-0.941) |
| External validation set Ⅰ | 0.902 (0.878-0.925) | 0.907 (0.873-0.940) | 0.935 (0.863-1.006) |
| External validation set Ⅱ | 0.938 (0.913-0.963) | 0.917 (0.883-0.951) | 0.913 (0.798-1.028) |
| **30-day readmission** |  |  |  |
| Training set | 0.934 (0.923-0.945) | 0.941 (0.928-0.953) | 0.967 (0.944-0.989) |
| Internal validation set | 0.932 (0.915-0.949) | 0.936 (0.916-0.955) | 0.950 (0.912-0.989) |
| External validation set Ⅰ | 0.845 (0.813-0.877) | 0.889 (0.848-0.930) | 0.896 (0.809-0.982) |
| External validation set Ⅱ | 0.935 (0.908-0.963) | 0.912 (0.873-0.951) | 0.973 (0.921-1.025) |

*Data in parentheses are 95% confidence intervals. LOS: length of stay; NPV: negative predictive value.*

**Table S7.** The PPVs in prolonged LOS and 30-day readmission models of different age subgroups.

| **Models** | **Group1 (<75 years)** | **Group2 (75~84 years)** | **Group3 (>84 years)** |
| --- | --- | --- | --- |
| **Prolonged LOS** |  |  |  |
| Training set | 0.521 (0.487-0.556) | 0.492 (0.455-0.529) | 0.490 (0.412-0.569) |
| Internal validation set | 0.515 (0.462-0.568) | 0.487 (0.428-0.547) | 0.506 (0.395-0.618) |
| External validation set Ⅰ | 0.490 (0.408-0.571) | 0.380 (0.294-0.467) | 0.486 (0.320-0.651) |
| External validation set Ⅱ | 0.319 (0.209-0.429) | 0.377 (0.262-0.491) | 0.323 (0.158-0.487) |
| **30-day readmission** |  |  |  |
| Training set | 0.372 (0.336-0.408) | 0.296 (0.254-0.337) | 0.348 (0.261-0.435) |
| Internal validation set | 0.380 (0.324-0.437) | 0.389 (0.304-0.474) | 0.400 (0.248-0.552) |
| External validation set Ⅰ | 0.332 (0.274-0.390) | 0.303 (0.236-0.369) | 0.303 (0.146-0.460) |
| External validation set Ⅱ | 0.150 (0.085-0.216) | 0.271 (0.191-0.351) | 0.294 (0.078-0.511) |

*Data in parentheses are 95% confidence intervals. LOS: length of stay; PPV: positive predictive value.*

**Table S8.** The brier scores in prolonged LOS and 30-day readmission models of different age subgroups.

| **Models** | **Group1 (<75 years)** | **Group2 (75~84 years)** | **Group3 (>84 years)** |
| --- | --- | --- | --- |
| **Prolonged LOS** |  |  |  |
| Training set | 0.170 (0.162-0.179) | 0.173 (0.163-0.181) | 0.173 (0.163-0.182) |
| Internal validation set | 0.163 (0.151-0.176) | 0.165 (0.152-0.178) | 0.170 (0.156-0.184) |
| External validation set Ⅰ | 0.119 (0.103-0.135) | 0.124 (0.102-0.146) | 0.145 (0.099-0.191) |
| External validation set Ⅱ | 0.085 (0.064-0.106) | 0.116 (0.090-0.141) | 0.153 (0.094-0.213) |
| **30-day readmission** |  |  |  |
| Training set | 0.106 (0.097-0.114) | 0.095 (0.085-0.105) | 0.094 (0.073-0.116) |
| Internal validation set | 0.104 (0.091-0.117) | 0.106 (0.093-0.120) | 0.119 (0.103-0.135) |
| External validation set Ⅰ | 0.160 (0.143-0.176) | 0.149 (0.127-0.171) | 0.118 (0.073-0.163) |
| External validation set Ⅱ | 0.072 (0.052-0.092) | 0.117 (0.093-0.142) | 0.065 (0.012-0.118) |

*Data in parentheses are 95% confidence intervals. LOS: length of stay.*

**Figure S1.** Comprehensive evaluation of the prolonged LOS model. **A-B** The ROC curves of the model in external validation set Ⅰ and external validation set Ⅱ. **C-D** The calibration curves of the model in external validation Ⅰ set and external validation set Ⅱ. The diagonal dotted line indicates the best prediction by an ideal model. The apparent line represents the uncorrected performance of the nomogram, and the red line shows the bias-corrected performance. **E-F** The decision curves of the model in external validation Ⅰ set and external validation set Ⅱ. The net benefits were measured at different threshold probabilities. The red line represents the identification nomogram. The gray line represents the assumption that all patients are identified as prolonged LOS. The black line represents the assumption that no patients are identified as prolonged LOS. **G-H** The clinical impact curves of the model in external validation Ⅰ set and external validation set Ⅱ. Clinical impact curve to identify prolonged LOS for a population size of 1000. The blue curve shows the identified number of prolonged LOS at different threshold probabilities, and the red curve represents actual number of prolonged LOS.


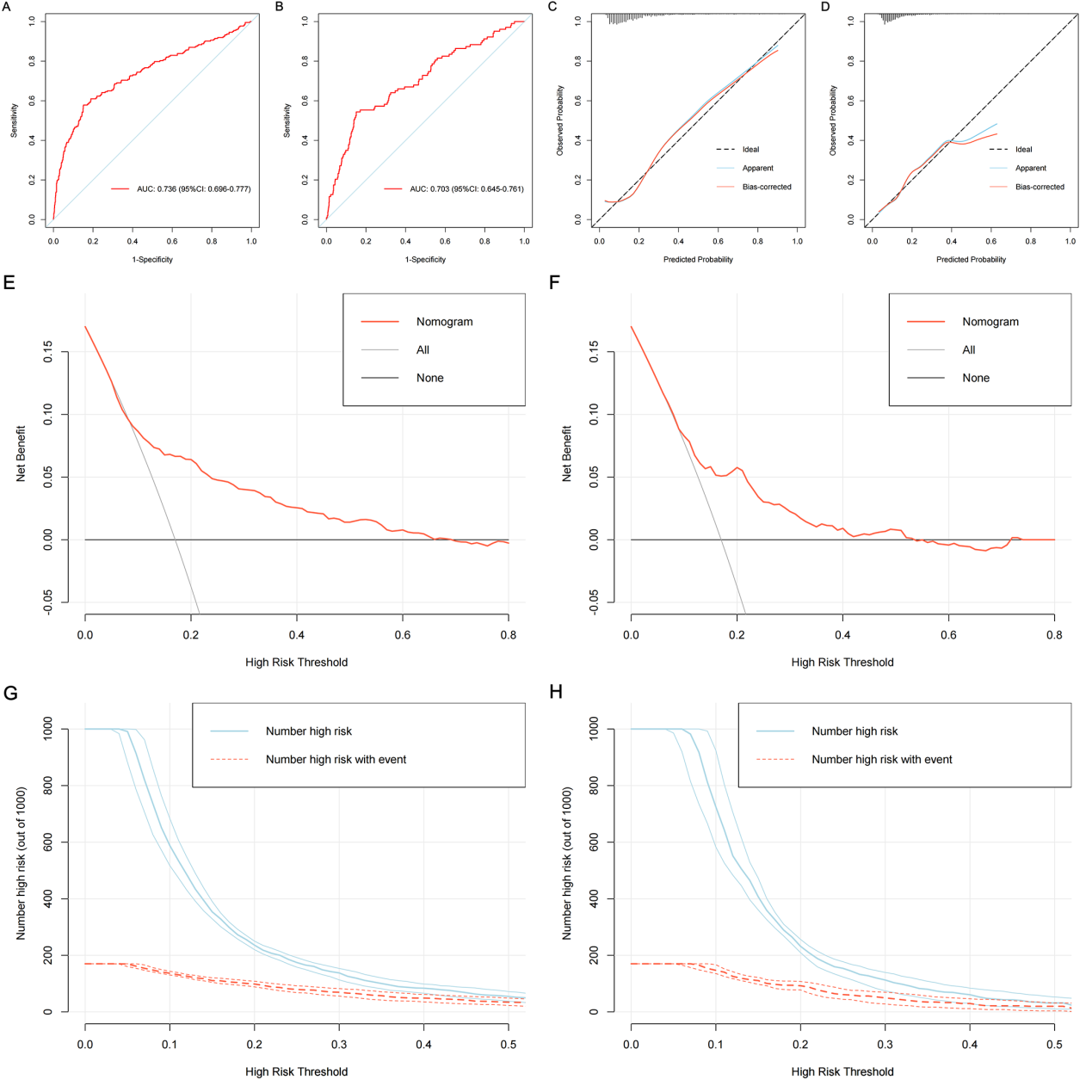


**Figure S2.** Comprehensive evaluation of the prolonged LOS model. **A-B** The decision curves of the model in training and internal validation sets. The net benefits were measured at different threshold probabilities. The red line represents the identification nomogram. The gray line represents the assumption that all patients are identified as prolonged LOS. The black line represents the assumption that no patients are identified as prolonged LOS. **C-D** The clinical impact curves of the model in training and internal validation sets. Clinical impact curve to identify prolonged LOS for a population size of 1000. The blue curve shows the identified number of prolonged LOS at different threshold probabilities, and the red curve represents actual number of prolonged LOS.

**
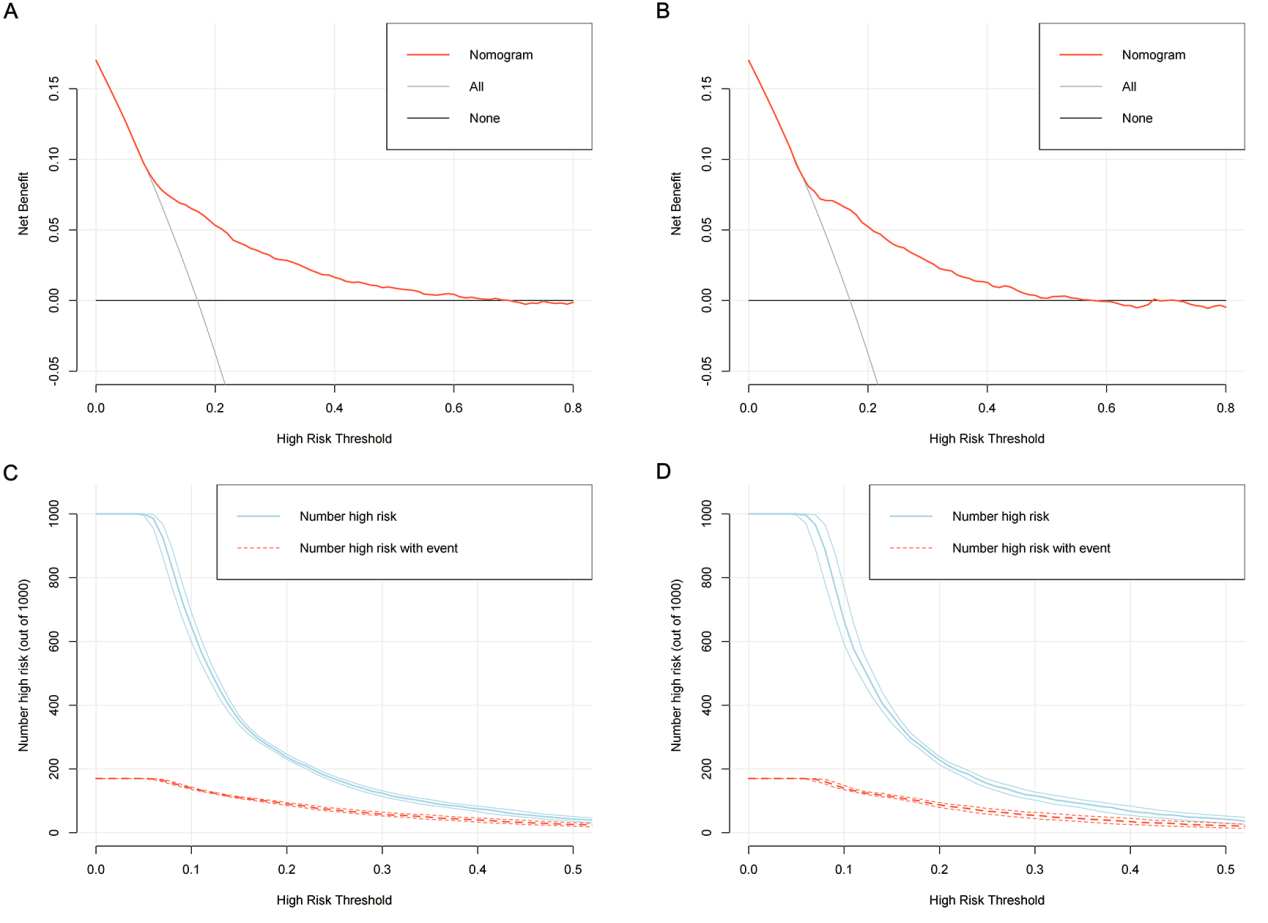
**

**Figure S3.** Comprehensive evaluation of the 30-day readmission model. **A-B** The ROC curves of the model in external validation Ⅰ set and external validation set Ⅱ. **C-D** The calibration curves of the model in external validation Ⅰ set and external validation set Ⅱ. The diagonal dotted line indicates the best prediction by an ideal model. The apparent line represents the uncorrected performance of the nomogram, and the red line shows the bias-corrected performance. **E-F** The decision curves of the model in external validation Ⅰ set and external validation set Ⅱ. The net benefits were measured at different threshold probabilities. The red line represents the identification nomogram. The gray line represents the assumption that all patients are identified as 30-day readmission. The black line represents the assumption that no patients are identified as 30-day readmission. **G-H** The clinical impact curves of the model in external validation Ⅰ set and external validation set Ⅱ. Clinical impact curve to identify 30-day readmission for a population size of 1000. The blue curve shows the identified number of 30-day readmission at different threshold probabilities, and the red curve represents actual number of 30-day readmission.


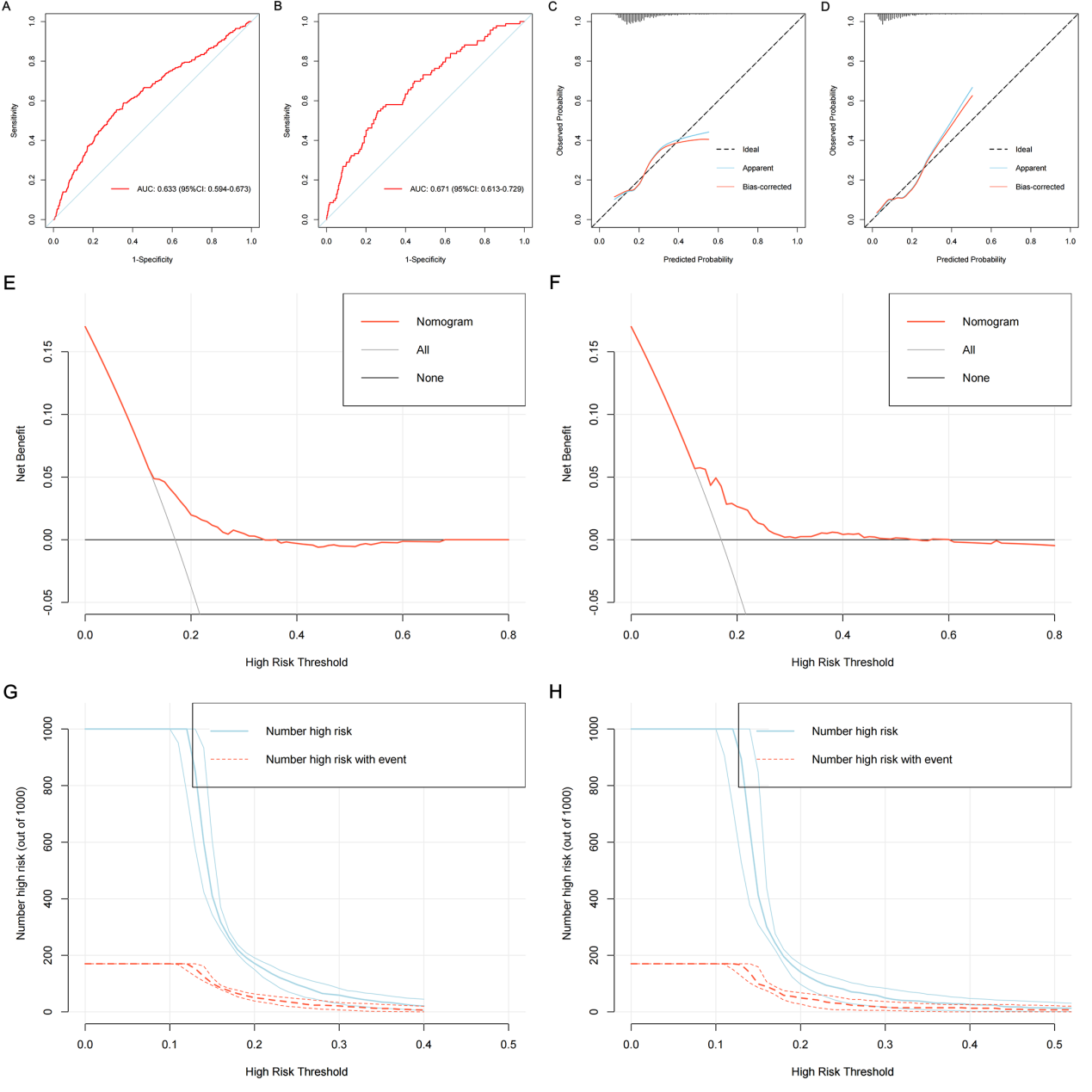


**Figure S4.** Comprehensive evaluation of the 30-day readmission model. **A-B** The decision curves of the model in training and internal validation sets. The net benefits were measured at different threshold probabilities. The red line represents the identification nomogram. The gray line represents the assumption that all patients are identified as 30-day readmission. The black line represents the assumption that no patients are identified as 30-day readmission. **C-D** The clinical impact curves of the model in training and internal validation sets. Clinical impact curve to identify 30-day readmission for a population size of 1000. The blue curve shows the identified number of 30-day readmission at different threshold probabilities, and the red curve represents actual number of 30-day readmission.

**
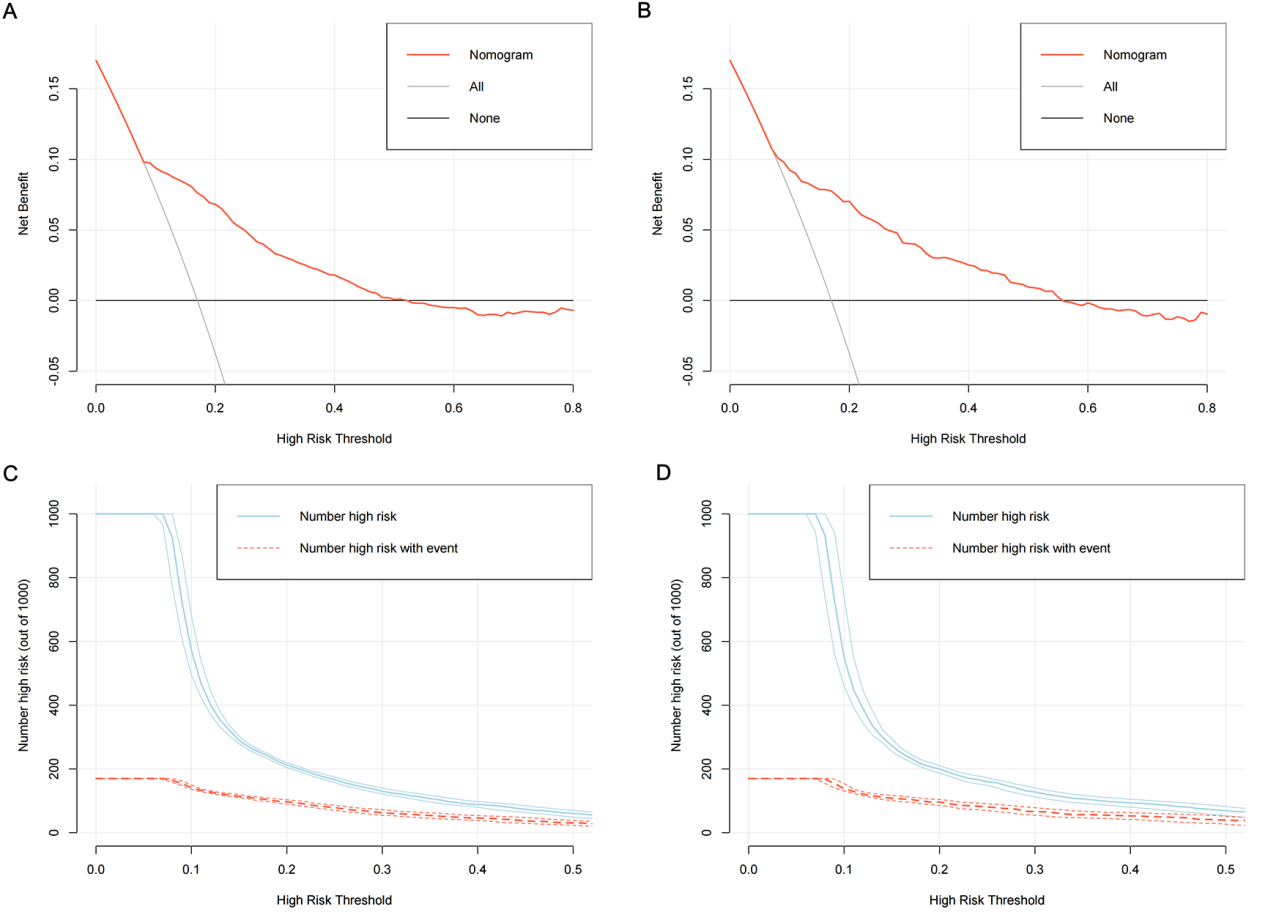
**

**Figure S5.** Comprehensive evaluation of age subgroups in the prolonged LOS model of the training set. **A** ROC curves. **B-D** The calibration curves of the <75, 75-84, and >84 groups. **E-G** The decision curves of the <75, 75-84, and >84 groups. **H-J** The clinical impact curves of the <75, 75-84, and >84 groups.


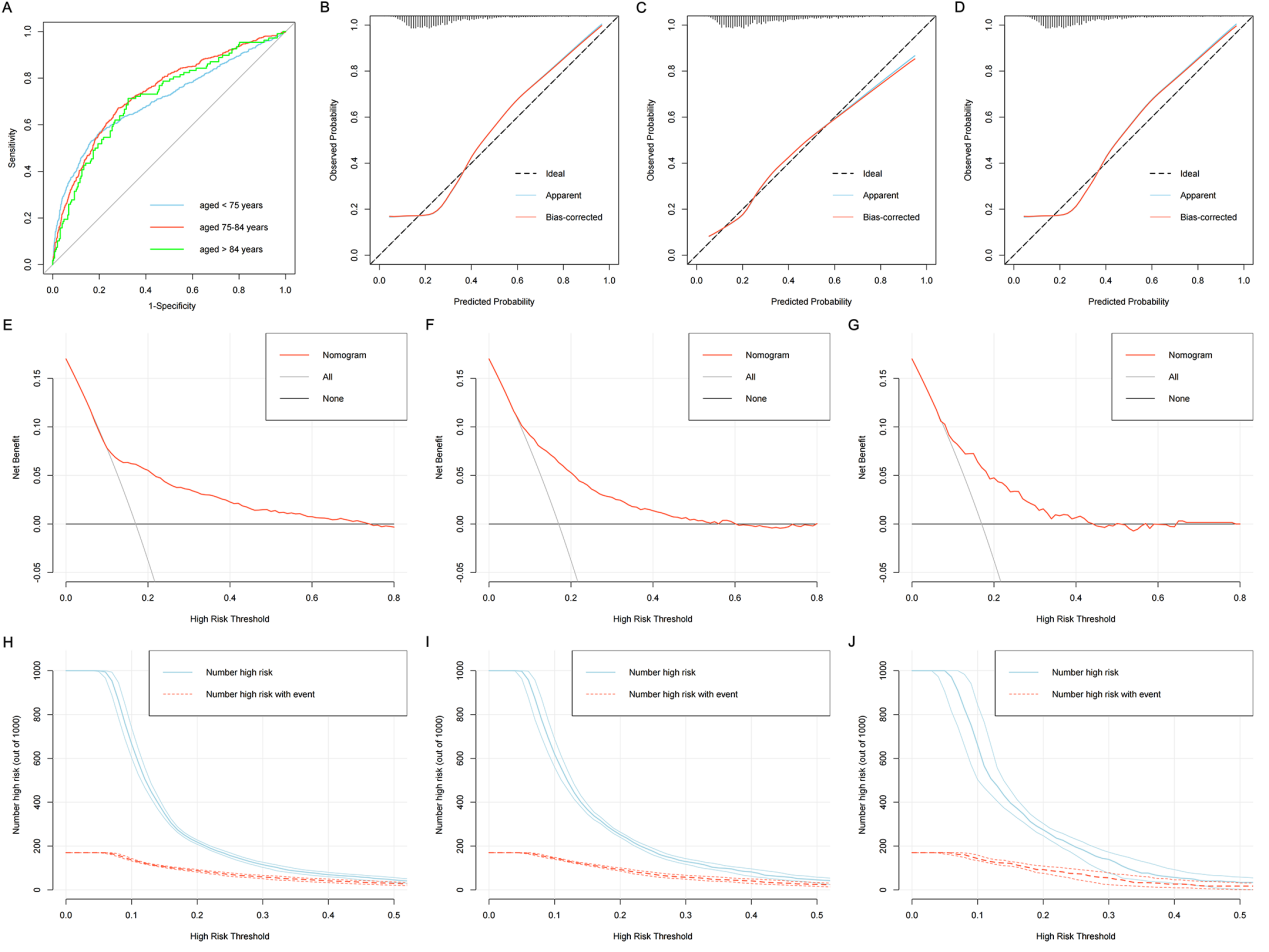


**Figure S6.** Comprehensive evaluation of age subgroups in the prolonged LOS model of the internal validation set. **A** ROC curves. **B-D** The calibration curves of the <75, 75-84, and >84 groups. **E-G** The decision curves of the <75, 75-84, and >84 groups. **H-J** The clinical impact curves of the <75, 75-84, and >84 groups.


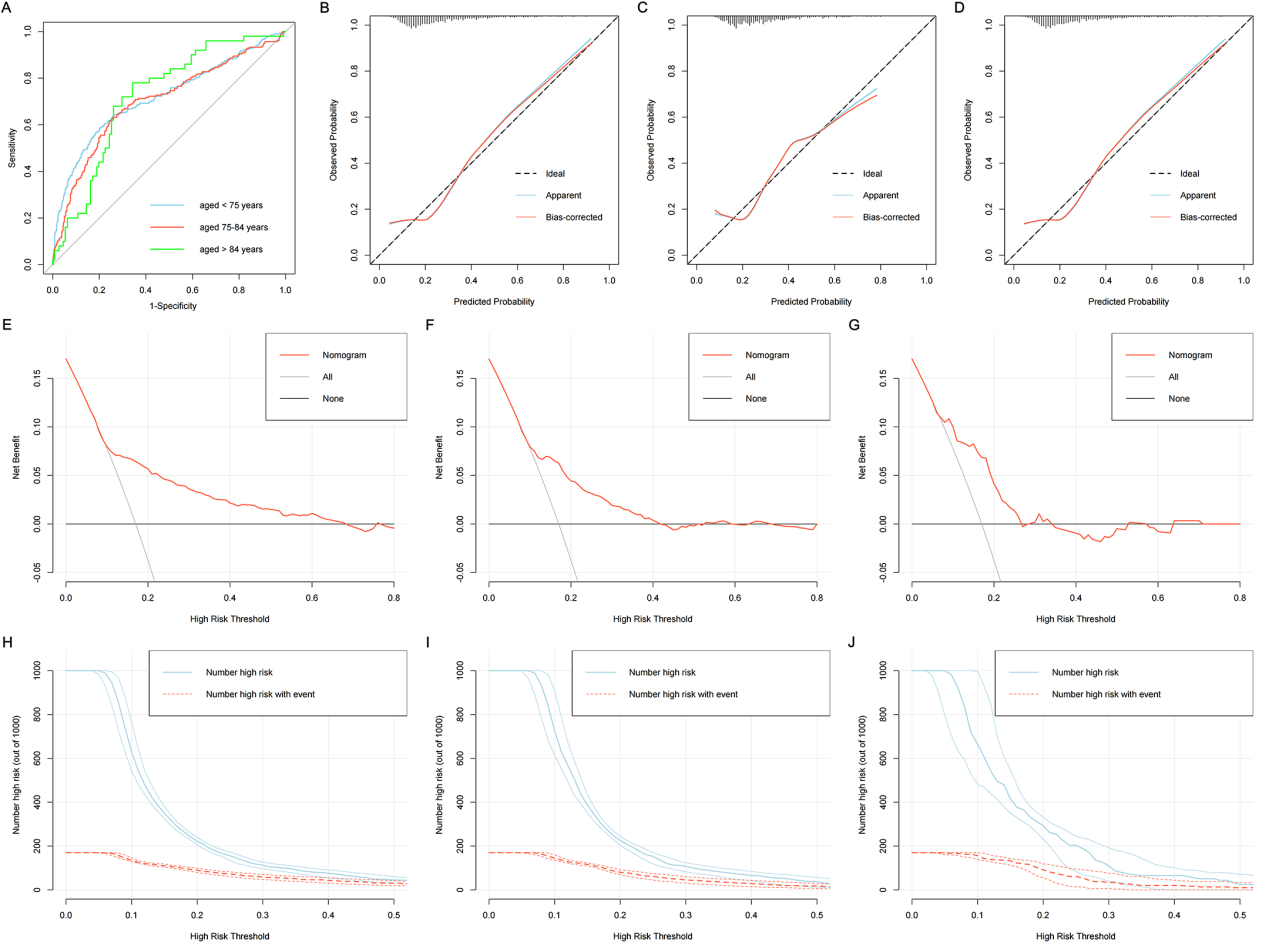


**Figure S7.** Comprehensive evaluation of age subgroups in the prolonged LOS model of the external validation set Ⅰ. **A** ROC curves. **B-D** The calibration curves of the <75, 75-84, and >84 groups. **E-G** The decision curves of the <75, 75-84, and >84 groups. **H-J** The clinical impact curves of the <75, 75-84, and >84 groups.


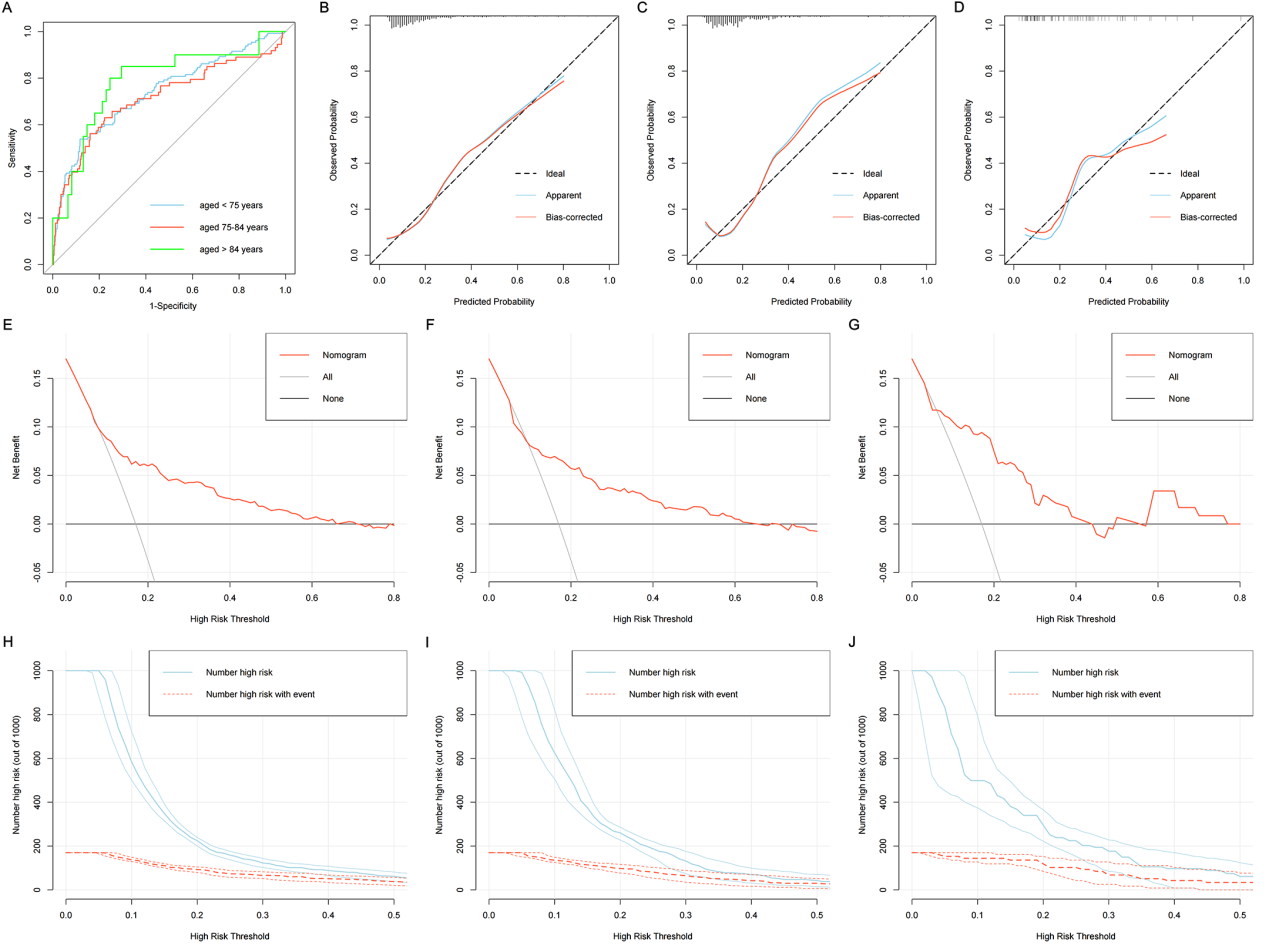


**Figure S8.** Comprehensive evaluation of age subgroups in the prolonged LOS model of the external validation set Ⅱ. **A** ROC curves. **B-D** The calibration curves of the <75, 75-84, and >84 groups. **E-G** The decision curves of the <75, 75-84, and >84 groups. **H-J** The clinical impact curves of the <75, 75-84, and >84 groups.


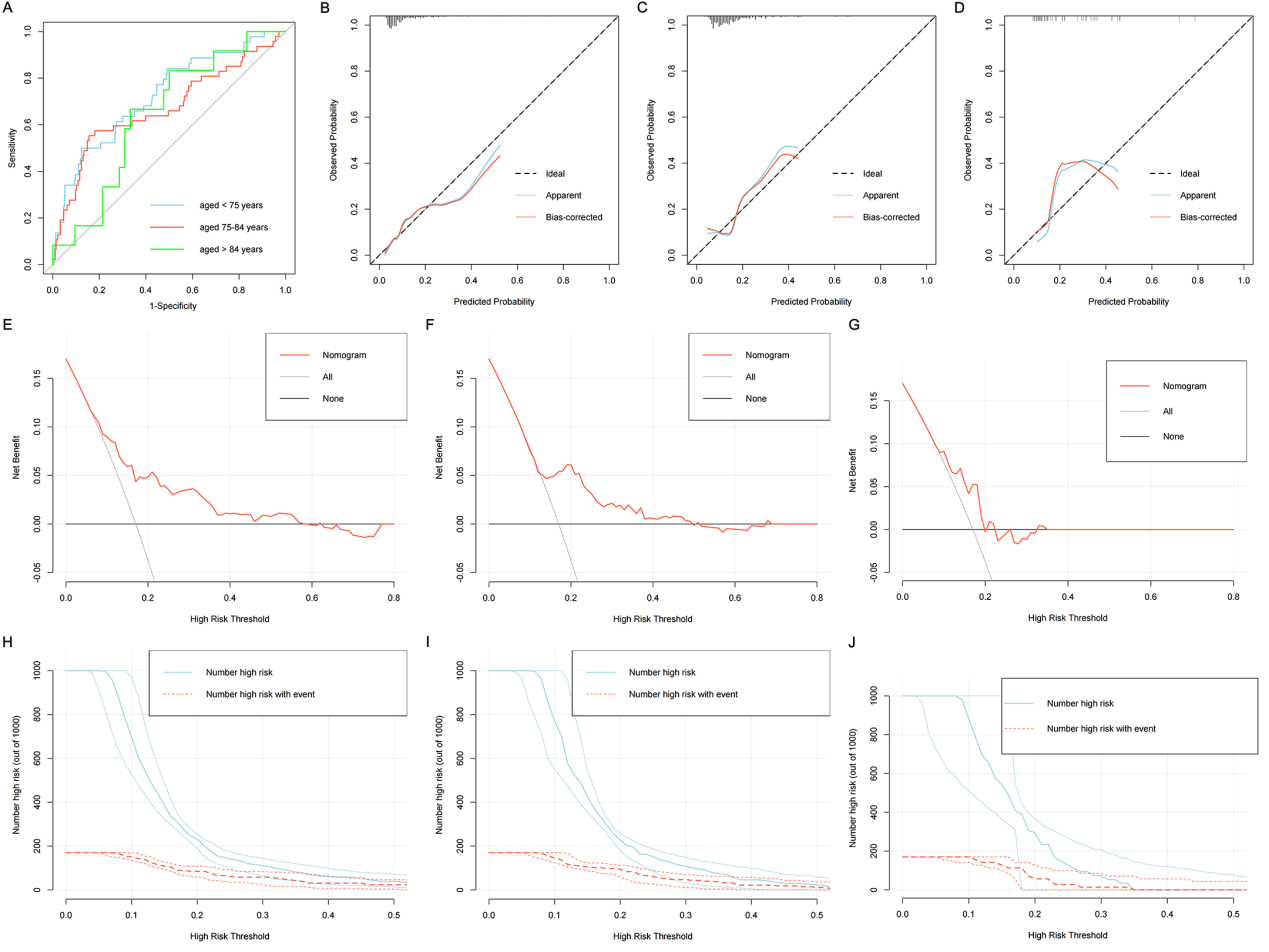


**Figure S9.** Comprehensive evaluation of age subgroups in the 30-day readmission model of the training set. **A** ROC curves. **B-D** The calibration curves of the <75, 75-84, and >84 groups. **E-G** The decision curves of the <75, 75-84, and >84 groups. **H-J** The clinical impact curves of the <75, 75-84, and >84 groups.


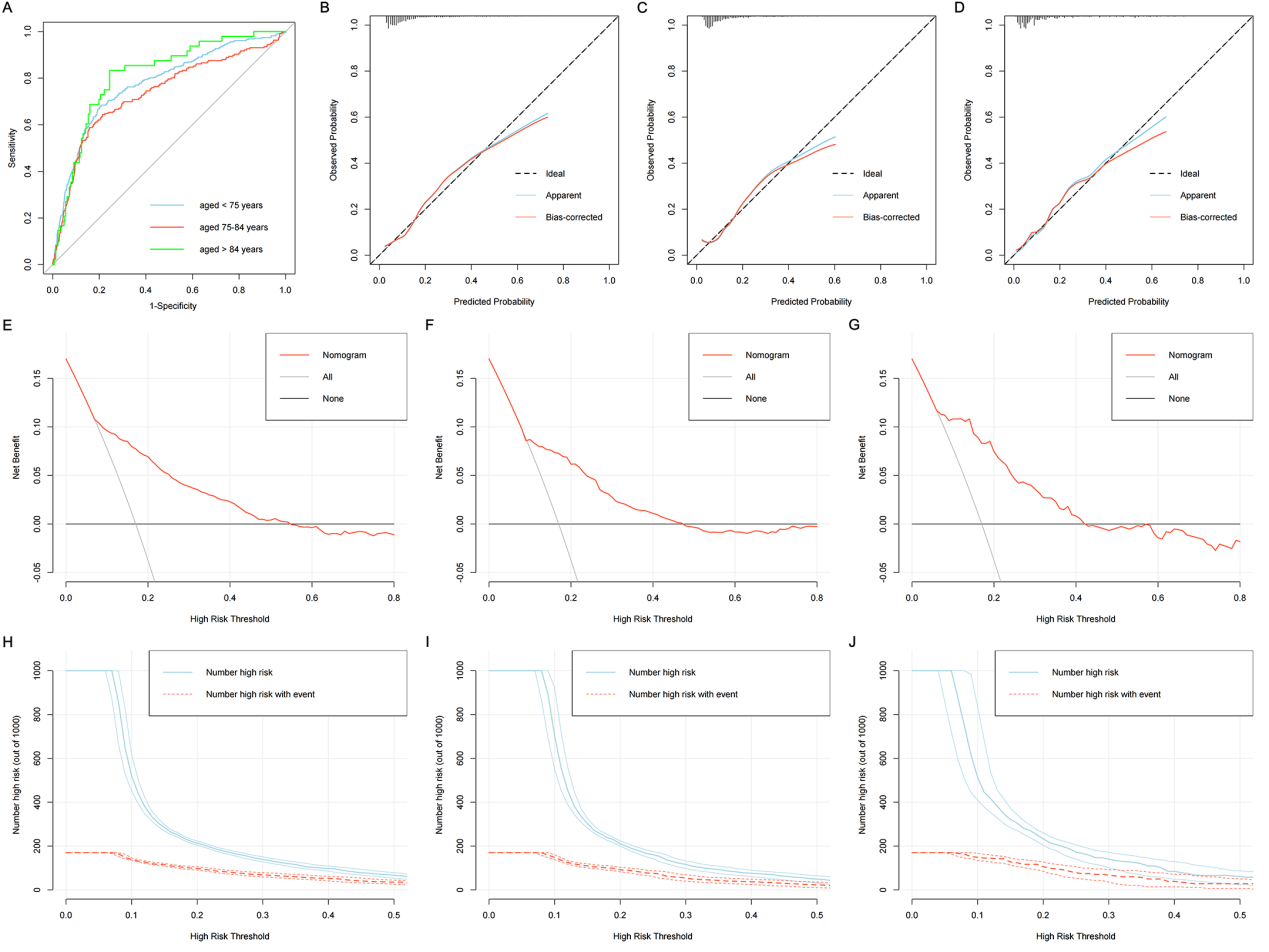


**Figure S10.** Comprehensive evaluation of age subgroups in the 30-day readmission model of the internal validation set. **A** ROC curves. **B-D** The calibration curves of the <75, 75-84, and >84 groups. **E-G** The decision curves of the <75, 75-84, and >84 groups. **H-J** The clinical impact curves of the <75, 75-84, and >84 groups.


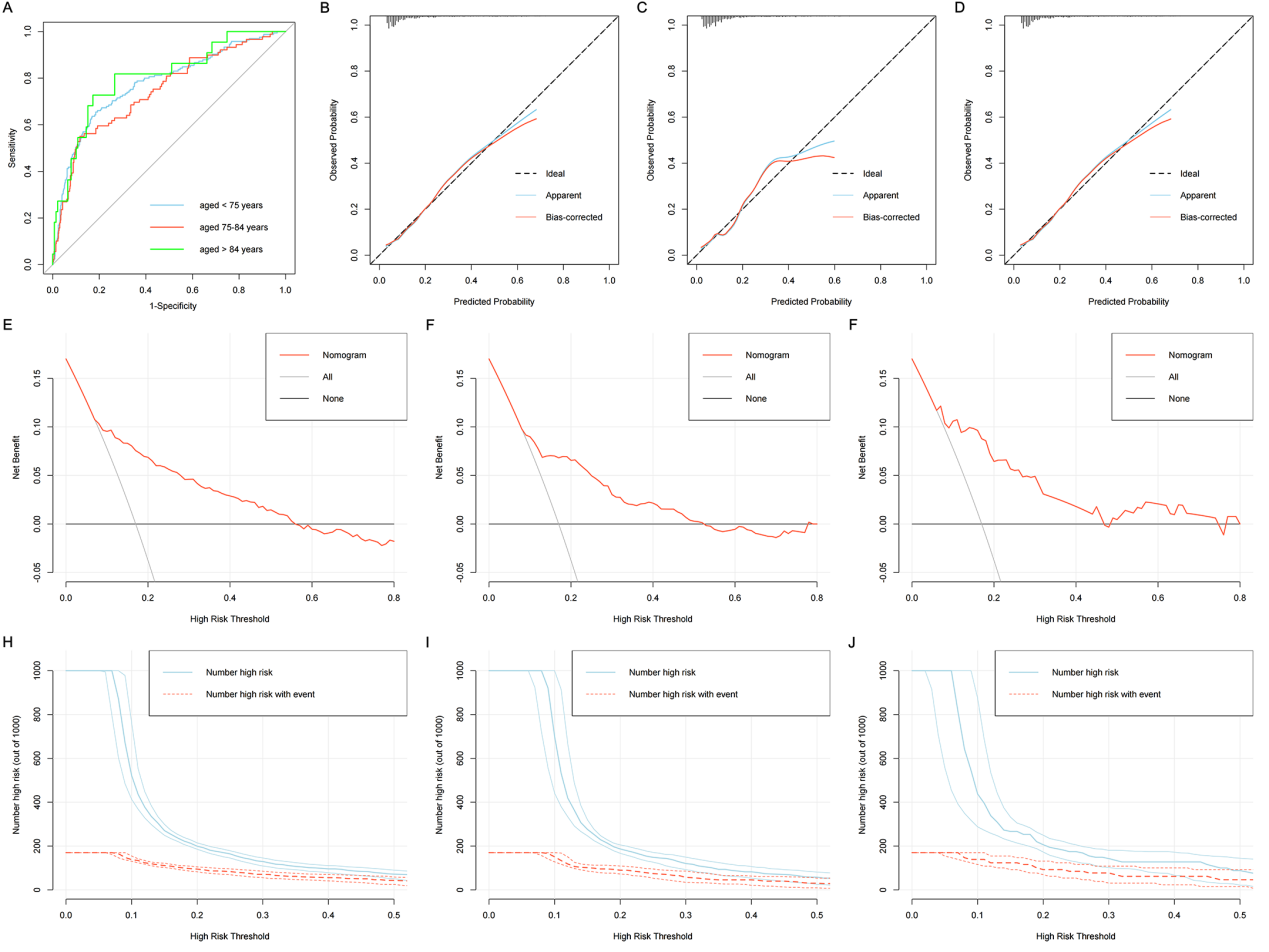


**Figure S11.** Comprehensive evaluation of age subgroups in the 30-day readmission model of the external validation set Ⅰ. **A** ROC curves. **B-D** The calibration curves of the <75, 75-84, and >84 groups. **E-G** The decision curves of the <75, 75-84, and >84 groups. **H-J** The clinical impact curves of the <75, 75-84, and >84 groups.


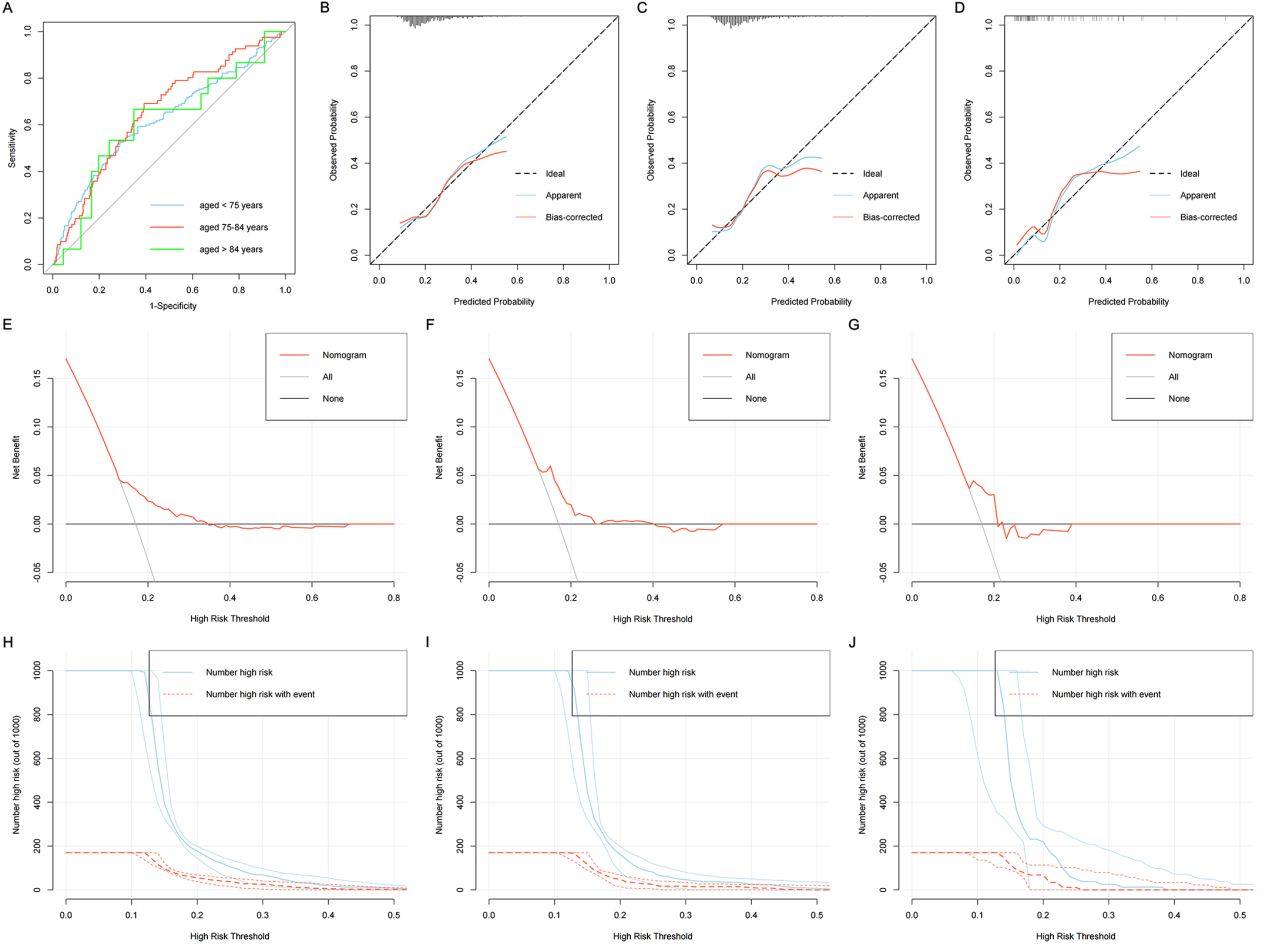


**Figure S12.** Comprehensive evaluation of age subgroups in the 30-day readmission model of the external validation set Ⅱ. **A** ROC curves. **B-D** The calibration curves of the <75, 75-84, and >84 groups. **E-G** The decision curves of the <75, 75-84, and >84 groups. **H-J** The clinical impact curves of the <75, 75-84, and >84 groups.


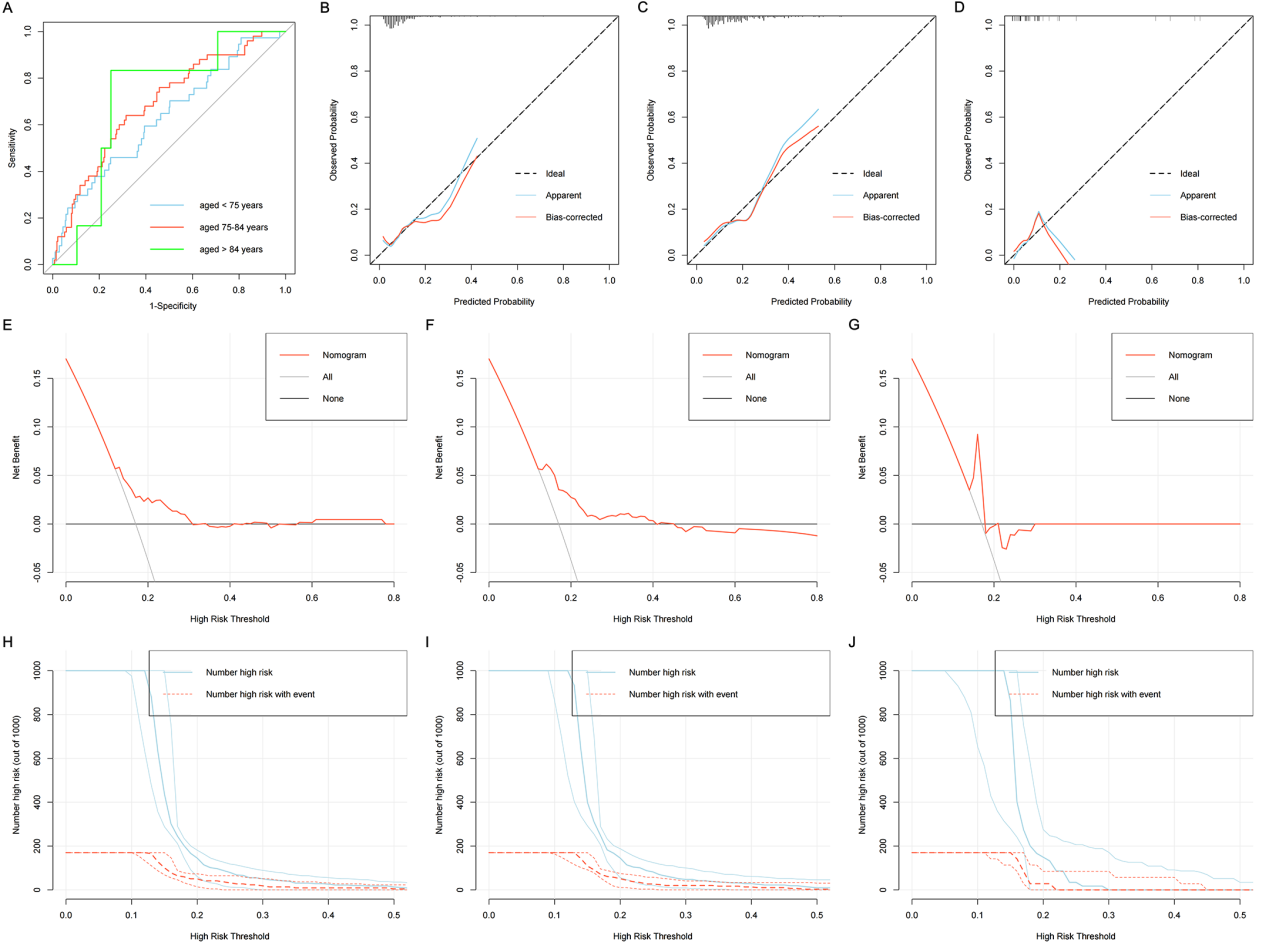

Supplement: Supporting Information — Additional supporting information can be found online in the Supporting Information section. This section includes a comparison of the datasets before and after multiple imputation; predictive performance metrics for prolonged LOS and 30-day readmission models, including sensitivity, specificity, NPV, PPV, and brier score; model evaluation plots, such as the ROC curve, calibration curve, decision curve, and clinical impact curve; and subgroup analysis results stratified by age. [file 3148242.f1.docx]
